# Supplementary material for: LIS1 RNA-binding orchestrates the mechanosensitive properties of embryonic stem cells in AGO2-dependent and independent ways
Source: Nat Commun. 2023 Jun 6;14:3293. doi: 10.1038/s41467-023-38797-8 (PMC10244377; doi:10.1038/s41467-023-38797-8)
Supplement: Supplementary file 1 — Supplementary Information [file 41467_2023_38797_MOESM1_ESM.pdf]

Supplementary Table 1- Media Compositions

**A. NHSM**			
DMEM-F12	240ml	L-ascorbic acid-2-phosphate	50ug/ml
Neurobasal	240ml	**Human LIF**	**20ng/ml**
Pen-strep 100X	5ml	**FGF2**	**8ng/ml**
GlutaMAX 100X	5ml	**TGFB1**	**1ng/ml**
NEAA 100X	5ml	**IWR1**	**5uM**
Human Insulin	12.5ug/ml	**Chir99021**	**3uM**
Apo-transferrin	100ug/ml	**PD0325901**	**1uM**
Progesterone	0.02ug/ml	**BIRB796**	**2uM**
Putrescine	16ug/ml	**SP600125**	**5uM**
Sodium Selenite	30nM	**LDN193189**	**0.4uM**
KSR	50ml	**Y27632**	**1.2uM**
B-mercaptoethanol (50 mM)	50ul		

**B. tHENSM**			
DMEM-F12	240ml	B-mercaptoethanol (50 mM)	50ul
Neurobasal	240ml	L-ascorbic acid-2-phosphate	50ug/ml
Pen-strep 100X	5ml	**Human LIF**	**20ng/ml**
GlutaMAX 100X	5ml	**XAV939**	**2uM**
NEAA 100X	5ml	**GO6983**	**2uM**
Human Insulin	12.5ug/ml	**CGP77675**	**1.2uM**
Apo-transferrin	100ug/ml	**DBZ**	**0.15uM**
Progesterone	0.02ug/ml	**PD0325901**	**0.33uM**
Putrescine	16ug/ml	**BIRB796**	**0.8uM**
Sodium Selenite	30nM	**Y27632**	**1.2uM**
B27	10ml	**Matrigel (reduced)**	**1 ml**
Dimethyl 2-oxoglutarate (aKG)	0.8mM (60ul)		

C. **<u>5i+LIF (mESCs)</u>**			
DMEM-F12	240ml	B-mercaptoethanol (50 mM)	50ul
Neurobasal	240ml	**Human LIF**	**20ng/ml**
Pen-strep 100X	5ml	**Chir99021**	**3uM**
GlutaMAX 100X	5ml	**GO6983**	**2uM**
NEAA 100X	5ml	**CGP77675**	**1.2uM**
N2 supplement 100x	5ml	**DBZ**	**0.15uM**
B27 +/- Vitamin A 50X	10ml	**PD0325901**	**0.33uM**
Dimethyl 2-oxoglutarate (aKG)	0.8mM (60ul)		

#XAV939 1uM was added, replacing CHIR99021 3uM to show WNT inhibition.

D. **<u>2i+LIF (mESCs)</u>**			
DMEM-F12	240ml	B27 +/- Vitamin A 50X	5ml
Neurobasal	240ml	Dimethyl 2-oxoglutarate (aKG)	0.8mM (60ul)
Pen-strep 100X	5ml	B-mercaptoethanol (50 mM)	50ul
GlutaMAX 100X	5ml	**Human LIF**	**20ng/ml**
NEAA 100X	5ml	**Chir99021**	**3uM**
N2 supplement 100x	5ml	**PD0325901**	**1uM**

E. **<u>Serum+LIF (mESCs)</u>**			
DMEM	Up to 500 ml	NEAA 100X	5ml
Fetal bovine serum	15ml	Dimethyl 2-oxoglutarate (aKG)	0.8mM (60ul)
Pen-strep 100X	5ml	B-mercaptoethanol (50 mM)	50ul
GlutaMAX 100X	5ml	**Human LIF**	**20ng/ml**

F. **<u>TNK-i + ERK-i + LIF (mESCs)</u>**			
DMEM-F12	240ml	B27 +/- Vitamin A 50X	5ml
Neurobasal	240ml	B-mercaptoethanol (50 mM)	50ul
Pen-strep 100X	5ml	Human LIF	20ng/ml

GlutaMAX 100X	5ml	**XAV939**	**1uM**
NEAA 100X	5ml	**PD0325901**	**1uM**
N2 supplement 100x	5ml		

**G. FGF+Activin (mESCs)**			
DMEM-F12	240ml	B27	10ml
Neurobasal	240ml	B-mercaptoethanol (50 mM)	50ul
Pen-strep 100X	5ml	**FGF2**	**12ng/ml**
NEAA 100X	5ml	**Activin-A**	**20ng/ml**
N2 supplement 100x	5ml		

DMEM/F-12, no glutamine, Gibco, Thermo Fisher Scientific, 21331020

Neurobasal medium (1X), Gibco, Thermo Fisher Scientific, 21103-049

Penicillin Streptomycin Solution, Biological Industries, 03-031-1B

GlutaMAX-I Supplement, 200mM, Thermo Fisher Scientific, 35050-038

NEAA, MEM Non-Essential Amino Acids Solution, Biological Industries, 01-340-1B

Insulin, Human Recombinant, Sigma Aldrich, 91077C-250MG

Apo-transferrin, Sigma Aldrich, T1147-1G

Progesterone, Sigma Aldrich, P8783-1G

Putrescine Dihydrochloride, Sigma Aldrich, P5780

Sodium Selenite, Sigma Aldrich, S5261-10G

KSR, KnockOut Serum Replacement, Gibco, Thermo Fisher Scientific, 10828-028

2-mercaptoethanol (1000X), Gibco, Thermo Fisher Scientific, 31350-010

Human LIF recombinant, PeproTech, 300-05

FGF2, recombinant human FGF-basic (146 a.a.), Peprotech, 100-18C

TGFB1, Recombinant Human, CytoLab Ltd. (Cytokines), PeproTech, 100-21C

IWR-1, MedChem Express, Biotag, HY-12238

Chir99021, MedChem Express, Biotag, HY-10182

PD0325901 (Mirdamenitib), MedChem Express, Biotag, HY-10254

BIRB796 (Doramapimod), MedChem Express, Biotag, HY-10320

SP600125, MedChem, Biotag, HY-12041

LDN193189 Hydrochloride, MedChem Express, Biotag, HY-12071A

Y-27632 Dihydrochloride, MedChem Express, Biotag, Hy-10583

B-27 Supplement minus Vitamin A (50X), Thermo Fisher Scientific, Rhenium, 12587

B-27 Serum-Free Supplement (50X), Thermo Fisher Scientific, Rhenium, 5017504044

Dimethyl 2-oxoglutarate (aKG), 96%, Sigma Aldrich, 349631

XAV-939, MedChem Express, Biotag, HY-15147

GO 6983, MedChem Express, Biotag, HY-13689

CGP77675, Sigma Aldrich, SML0314

DBZ, Tocric-Bio-Techne Corp, Biotest, 4489

Matrigel GF reduced, Corning Limited, FAL356231

N-2 supplement (100X), Thermo Fisher Scientific, Rhenium, 17502001

Fetal bovine serum, Qualified for hESCs, Biological Industries, 04-002-1A

Activin-A, recombinant, CytoLab Ltd. (Cytokies), PeproTech, 120-14E

Supplementary Table 2 - Primers for qPCR and genotyping

Quantitative RT-PCR

The miR cDNA for qRT experiments was prepared with miScript II RT kit (Qiagen, 218161) using HiFlex buffer. The qRT reactions were performed with miScript SYBR Green PCR kit (Qiagen, 218075). The values of miRNAs were normalized to that of U6 snRNA. The following primers were used for the reactions:

U6	5’-CTCGCTTCGGCAGCACA-3’
mmu-let7b-5p	5’-TGAGGTAGTAGGTTGTGTGGTT-3’
mmu-mir-181a-5p	5’-AACATTCAACGCTGTCGGTGACT-3’
mmu-mir-214-3p	5’-ACAGCAGGCACAGACAGGCAGT-3’
mmu-mir-221-3p	5’-AGCTACATTGTCTGCTGGGTTTC-3’
mmu-mir-222-3p	5’-AGCTACATCTGGCTACTGGGTCT-3’
mmu-mir-31-5p	5’-AGGCAAGATGCTGGCATAGCTG-3’
mmu-mir-10a-5p	5’-TACCCTGTAGATCCGAATTTGTG-3’
mmu-mir-146-5p	5’-TGAGAACTGAATTCCATAGGCT-3’
mmu-mir-151-5p	5’-TCGAGGAGCTCACAGTCTAGT-3’
mmu-mir-16-5p	5’-TAGCAGCACGTAAATATTGGCG-3’
mmu-mir-302a-3p	5’-TAAGTGCTTCCATGTTTTGGTGA-3’
mmu-mir-302c-3p	5’-AAGTGCTTCCATGTTTCAGTGG-3’
mmu-mir-302d-3p	5’-TAAGTGCTTCCATGTTTTAGTAG-3’
mmu-mir-30c-5p	5’-TGTAAACATCCTACACTCTCAGC-3’
mmu-mir-5099-3p	5’-TTAGATCGATGTGGTGCTCC-3’
mmu-mir-541-5p	5’-AAGGGATTCTGATGTTGGTCACACT-3’
mmu-mir-6539-3p	5’-GCACAGTGATGAACTCTGAGGGCT-3’
mmu-let7c-5p	5’-TGAGGTAGTAGGTTGTATGGTT-3’
mmu-let7a-5p	5’-TGAGGTAGTAGGTTGTATAGTT-3’
mmu-let7e-5p	5’-TGAGGTAGGAGGTTGTATAGTT-3’
mmu-let7i-5p	5’-TGAGGTAGTAGTTTGTGCTGTT-3’
mmu-miR-341-3p	5’ –TCGGTCGATCGGTCGGTCGGT- 3’

The reverse primer was miScript Universal Primer (Qiagen).

Quantitative PCR and preparation of cDNA for total RNA were performed with Fast SYBR Green Master Mix (Thermo Fisher 4385614) normalized to RPS29. The following primers were used for the reactions:

Rian-F	5'-TGTCACGGTCAGCTCTGTTC-3'
Rian-R	5'-ACCAAGGTGTACGCAACGAT-3'
RPS29-F	5'-TCGTTGGGCGTCTGAAGGCAA-3'
RPS29-R	5'CGGAAGCACTGGCGGCACAT-3'

Genotyping

Mice were genotyped at 21 days old by established methods using following primers:

129S-Pafah1b1^{tm2Awb}/J- (Lis1 $^{Flox/Flox}$, Lis1 $^{Flox/WT}$ or LIS1 $^{Flox/-}$):

Lis1 wild-type and floxed alleles were detected with

Forward primer 5'-GCTTCCTGTTCAGCAGATATG-3'

Reverse primer 5'-GCTTGTTCATCAAGCTTGCAC-3'

and deleted allele was detected with

Forward primer 5'-GGCGATGATAACCACTGAGTC -3'

Reverse primer 5'-GCTTGTTCATCAAGCTTGCAC-3'

B6-Tg(Pgk1-cre)1Lni and B6;129S-Tg(UBC-cre/ERT2)1Ejb/J :

Cre transgene was detected with

Forward primer 5'-TATCTTCTATATCTTCAGGCGC-3'

Reverse primer 5'-GTGAACGAACCTGGTCGAAATCA-3'

129S;ICR-*Tg(CAGG-loxP-LacZ-neo-loxP-PAFAH1B1-DsRed*):

Lis1-DsRed transgene was detected with

Forward primer 5'-ACCCTATGTCGTCACTGGCA-3'

Reverse Primer 5'-TGCTTCACGTACACCTTGGAG-3'

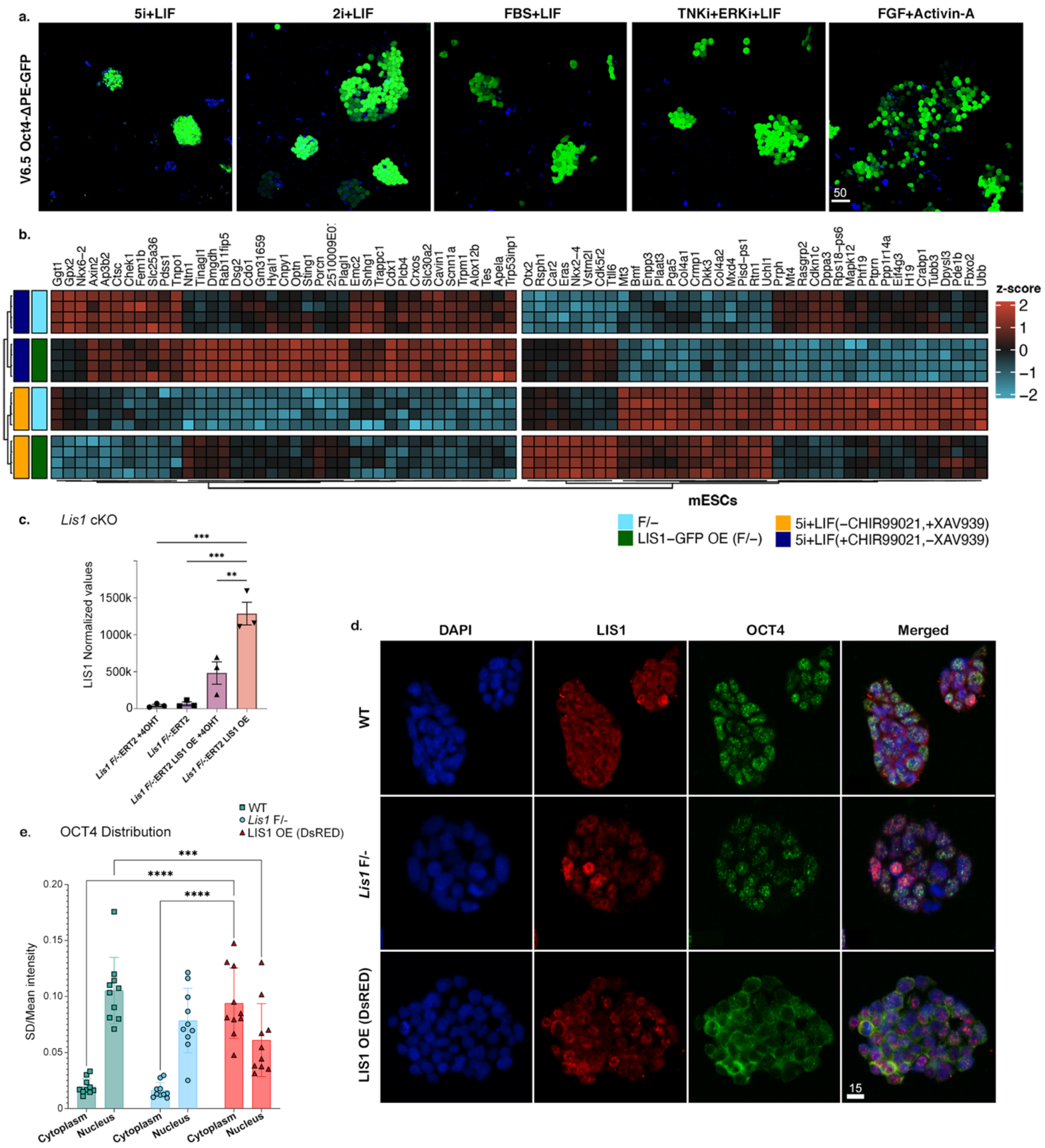

Supplementary Fig. 1**: WNT signaling influences the sustenance of mutant LIS1 embryonic stem cells. a)** Fluorescent images of V6.5 Oct4-ΔPE-GFP mESCs cultured using different media. Left to right: 5i+LIF, 2i+LIF, FBS+LIF, Tnki+Erki+LIF, and FGF+Activin; scale bars, 50 µm. **b)** A heatmap showing LIS1 dose-dependent gene expression changes common between mESC isogenic lines *Lis1* flox/- (F/-) and LIS1-GFP Overexpression on the background of *Lis1* F/- (LIS1-GFP OE (F/-)). Lines were cultures in two media conditions, 5i+LIF media, with the addition of either a WNT activator (CHIR99021) or a WNT inhibitor (XAV939). The data are shown on a Z-score scale of the variance stabilizing transformation on normalized reads. **c)** Normalized expression levels of LIS1 in isogenic mESCs lines, derived

from conditional mouse line expressing *Lis1* F/+ and ERT2:Cre (*Lis1* F/+ ERT2) treated or not treated with 4-Hydroxytamoxifen (4OHT). **d)** Representative images of immunostainings of mESCs quantified in **e**. stained with anti-OCT4 (green) and anti-LIS1 (red), scale bars, 15 µm. **e)** Nuclear (Nucleus) vs. Cytoplasmic (Cytoplasm) localization of OCT4 in mESCs isogenic lines *Lis1* flox/- (*Lis1* F/-), LIS1-DsRED overexpressing line (LIS1 OE) and wildtype (WT). For c. & d., data are presented as mean values ± SEM (n=3), One-way ANOVA, and Tukey's test for multiple comparisons, p-values: *p<0.05, **p<0.01, ***p<0.001, ****p<0.0001.

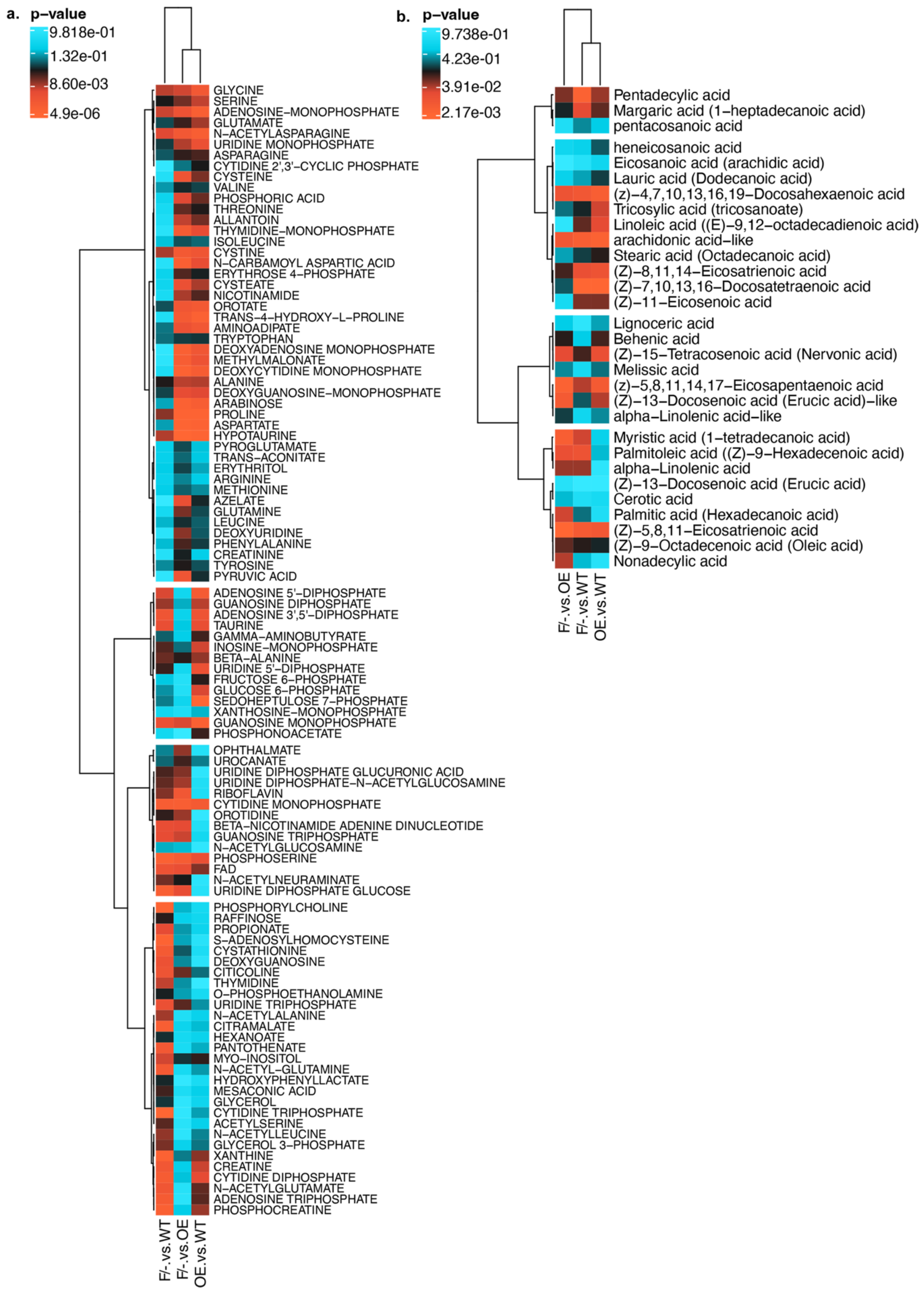

Supplementary Fig. 2: **LIS1 dosage affects the cellular metabolome. a)** Metabolomic analysis of mESCs with varying LIS1 levels. F/- vs. WT: Metabolites of *Lis1* flox/- (F/-) compared to that extracted from wildtype (WT). F/- vs. OE: F/- metabolites plotted against

LIS1-GFP overexpressing line (OE). OE vs. WT: Metabolites extracted from LIS1-GFP overexpressing line compared against WT metabolome. **b)** Comparative fatty acid metabolic profile of the same lines. For a. and b., a two-sided unpaired t-test was performed using two-tailed distribution and a two-sample equal variance (homoscedastic). There were no post hoc adjustments for multiple comparisons. The scale bar represents p-values.

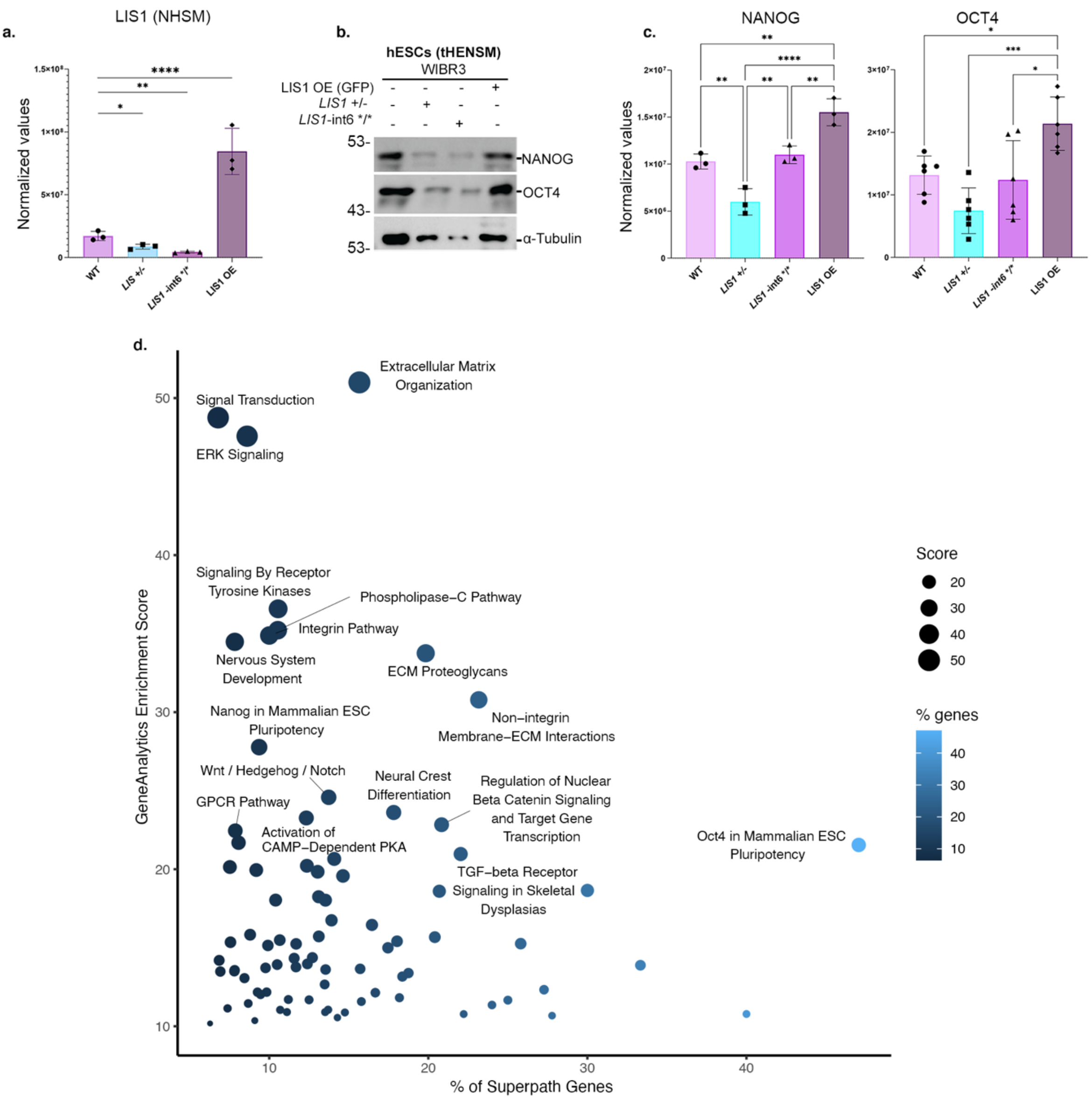

Supplementary Fig. 3 **LIS1 dosage affects the expression. a)** LIS1 dosage varies in four isogenic hESC lines. Normalized LIS1 levels (n=3, Data are presented as mean values ± SEM.) in WIBR3 (WT), *LIS1* heterozygous (*LIS1* -/+), and in a homozygous intronic mutation in intron 6 (*LIS1-int6*/**) and LIS1-GFP overexpressing line (LIS1-OE (GFP)). **b)** Western blot showing expression levels of the pluripotent transcription factors OCT4 and NANOG in the isogenic hESC lines with the indicated genotype. **c)** Expression levels were quantified from extracts from NANOG (n=3, Data are presented as mean values ± SEM.) and OCT4 (n=6). **d**) The GeneAnalytics pathway enrichment analysis for differentially expressed genes(n=897) between LIS1 +/- and LIS1 overexpression. Top significant pathways identified by GeneAnalytics above the enrichment score of twenty are shown in the plot.

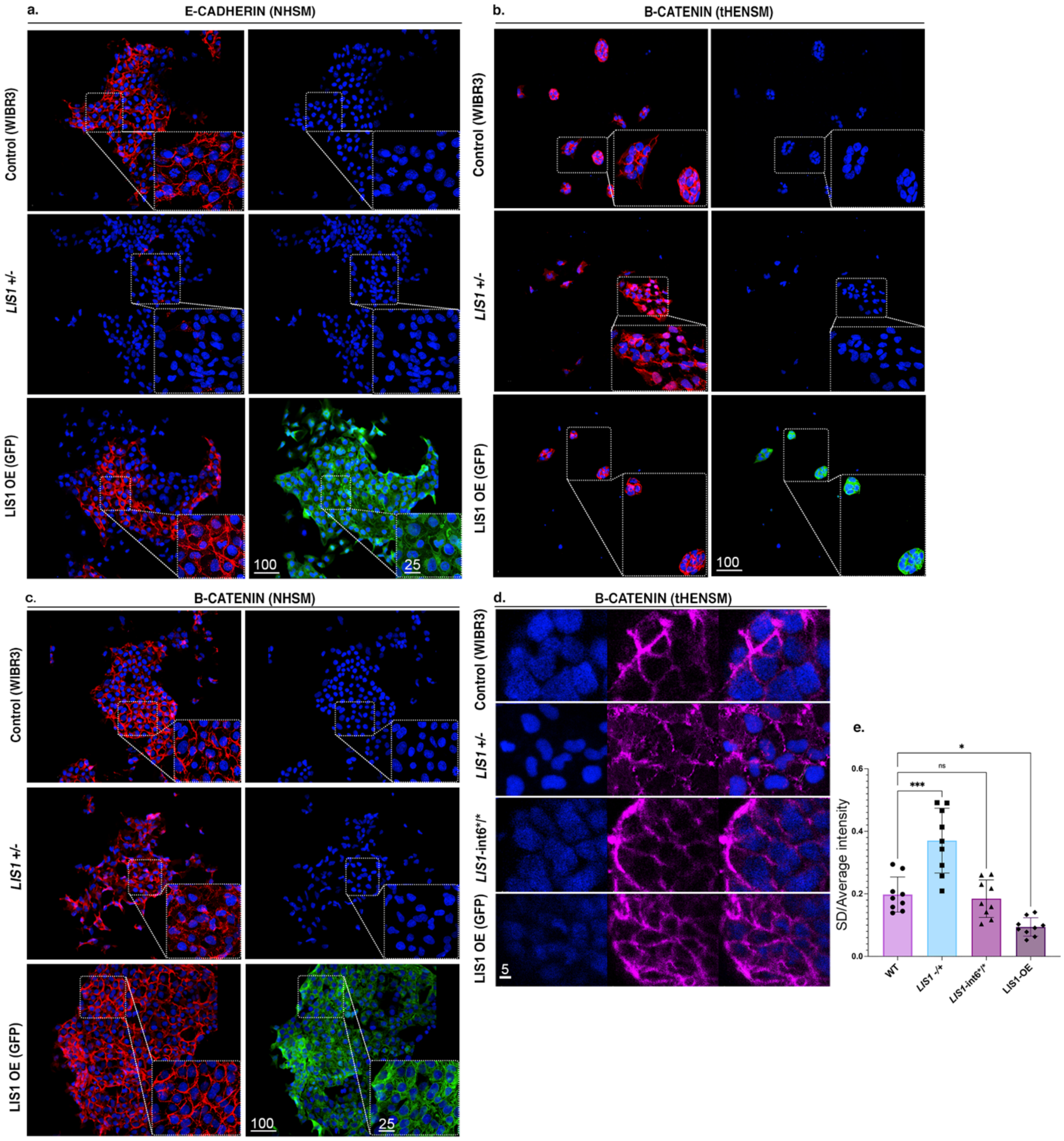

Supplementary Fig. 4 **LIS1 dosage affects the localization of pluripotency markers.**
a-c) Immunostaining of hESC lines (genotypes indicated) cultured in tHENSM or NHSM media. E-CADHERIN (**a**, red), B-CATENIN (**b,c** red). Scale bars, 100 µm. Insets represent a 2.5x zoom, scale bar, 25 µm. **d**) Images showing changes in B-CATENIN localization in hESC colonies (genotypes indicated). B-CATENIN (magenta) DAPI (blue). Scale bar, 5 µm. **e**) Quantification of the Standard deviation (SD) of B-CATENIN immunostaining intensity over the average intensity measured along comparable sections reflects the degree of uniform distribution of the protein at the cell membrane. n=9, Data are presented as mean values ± SD

One-way ANOVA and Dunnet's test for multiple comparisons, p-values: ns-not significant, * $p<0.05$ *** $p<0.001$.

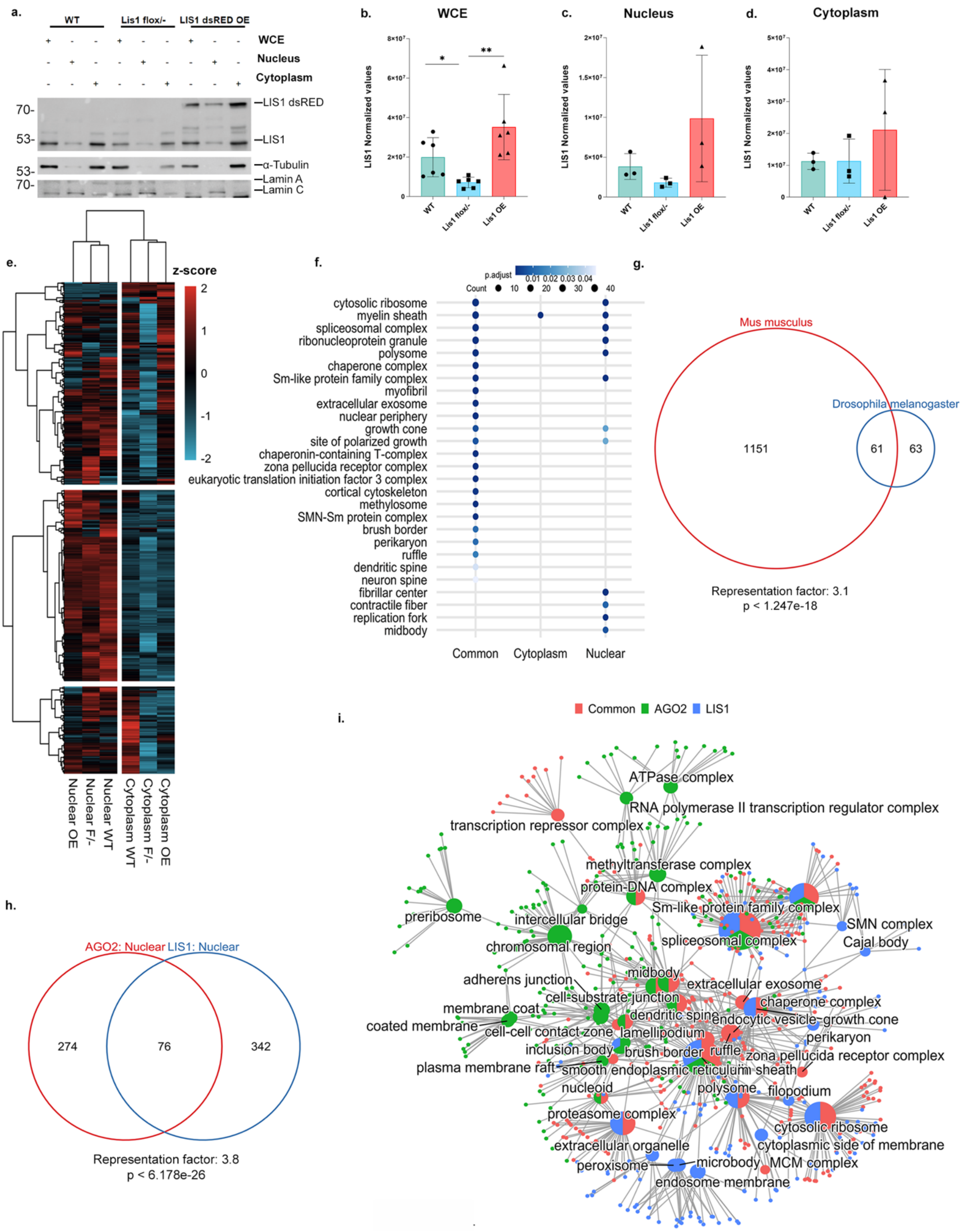

Supplementary Fig. 5**: The LIS1 interactome. a**) Biochemical fractionation of isogenic mESCs lines: Wildtype (WT), *Lis1* flox/- (*Lis1* F/-), and LIS1-DsRED overexpressing line (LIS1 OE). Western blots reacted with anti-LIS1, anti-α-Tubulin and anti-LAMIN A/C antibodies. **b-d**) Quantification of LIS1 localization in the different cell lines reveals

significant changes in the expression levels (WCE, whole cell extract, **b**) but not in the relative distribution in other compartments (Nucleus, **c**., Cytoplasm, **d**., n=3, Data are presented as mean values ± SEM.). One-way ANOVA and Tukey's test for multiple comparisons was performed, p-values: * $p<0.05$ **, $p<0.01$, *** $p<0.001$, ****$p<0.0001$. **e**) A heatmap of normalized peptide intensity on a log2 scale of proteins immunoprecipitated with anti-LIS1 antibodies using nuclear and cytoplasmic fractions from LIS1-dsRED overexpression (OE), wildtype (WT), or *Lis1* F/- (F/-) lines, identified by mass spectrometry, after scaling. **f**) Overrepresentation test analysis for GO terms of LIS1 interacting proteins. Categorized as common for proteins found in nuclear and cytoplasmic fractions, nuclear only, or cytoplasmic only. Enrichment was performed using the clusterProfiler package in R. P-values for enriched GO terms were adjusted for multiple comparisons by the Benjamini-Hochberg method, and terms with adjusted $P<0.05$ were considered significant. **g**) A Venn diagram showing the significant overlap of LIS1 interacting proteins identified in this study (all fractions/extracts) and *Drosophila melanogaster* orthologs from Guruharsha *et al*.[1] **h**) A Venn diagram showing the significant overlap of LIS1 interacting proteins identified in nuclear extracts of LIS1-dsRED OE, WT, and F/- with AGO2 nuclear-interacting proteins from Sharshad *et al*[2]. For **g**. and **h**. p-value < 0.05, one-sided hypergeometric test. **i**) The network of GO terms showing linkage for LIS1 interacting proteins from this study and AGO2 interacting proteins from Sharshad *et al*[2]. Big hub nodes with pie represent GO terms for the distribution of proteins specific to LIS1 (blue), AGO2 (green), and common (red) interacting proteins between the two. Pie size corresponds to the total number of proteins in each GO term. The small nodes represent proteins linked to GO term nodes.

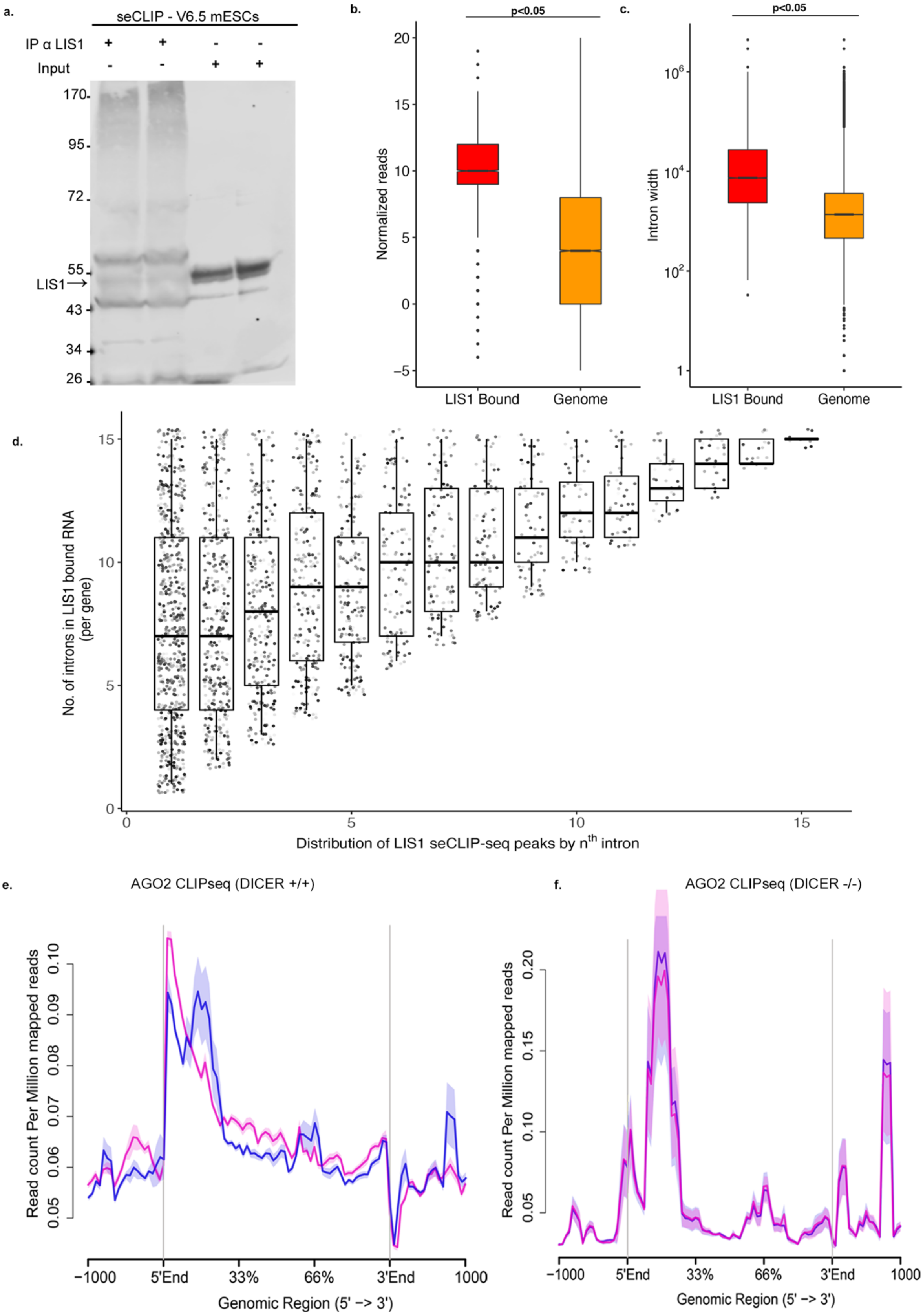

a.
seCLIP - V6.5 mESCs
IP α LIS1
Input
170
95
72
55
LIS1
43
34
26
b.
p<0.05
Normalized reads
LIS1 Bound
Genome
c.
p<0.05
Intron width
LIS1 Bound
Genome
d.
No. of introns in LIS1 bound RNA (per gene)
Distribution of LIS1 seCLIP-seq peaks by nth intron
e.
AGO2 CLIPseq (DICER +/+)
Read count Per Million mapped reads
Genomic Region (5' -> 3')
5'End
33%
66%
3'End
f.
AGO2 CLIPseq (DICER -/-)
Read count Per Million mapped reads
Genomic Region (5' -> 3')

Supplementary Fig. 6: **Combinatorial regulation of pre-mRNA processing by LIS1. a**) A western blot of LIS1 immunoprecipitation from V6.5 mESCs in the seCLIP experiment (two biological replicates). **b**) Expression level of LIS1-bound genes compared to the whole genome; two-sided unpaired t-test, P-value: *$p<0.05$. Values are log2 (baseMean) as calculated by DESeq2 (n=4 biological replicates). Boxplots show median and lower or upper quartiles; whiskers show inner fences. **c**) A boxplot of LIS1 bound intron (n=29721) width versus the entire genome introns (n=303499, Gencode vM25); two sided Wilcoxon ranked sum test, P value: *$p<0.05$. Boxplots show median and lower or upper quartiles; whiskers show inner fences. **d**) LIS1 seCLIP-seq clusters as a function of intron number within a gene (x-axis) and the total number of introns (1-15 introns) in LIS1 bound genes (y-axis). **e, f**) Metagene plot for the mean (± SEM) read coverage of AGO2 CLIP-seq data from the study of Leung *et al.*[3] in (**e**) WT embryonic stem cells DICER(+/+) and (**f**) DICER knockout embryonic stem cells. Statistics for 5b,c are in Supplementary Data 7A.

a.

MAJIQ	OE vs F/-
Alternative first exon	56
Alternative Intron	99
Alternative last exon	23
Alternative 3' & 5'-ss	8
Alternative 3'-ss	19
Alternative 5'-ss	33
Cassette exon	137
Mutually exclusive exon	6
other	53
putative alternative 3' or 5'-ss	18
putative alternative first exon	12
putative alternative last exon	12
**rMATS**	**OE vs F/-**
Alternative 3'-SS	145
Alternative 5'-SS	134
Mutually exclusive exon	196
Retained intron	242
Skipped exon	657

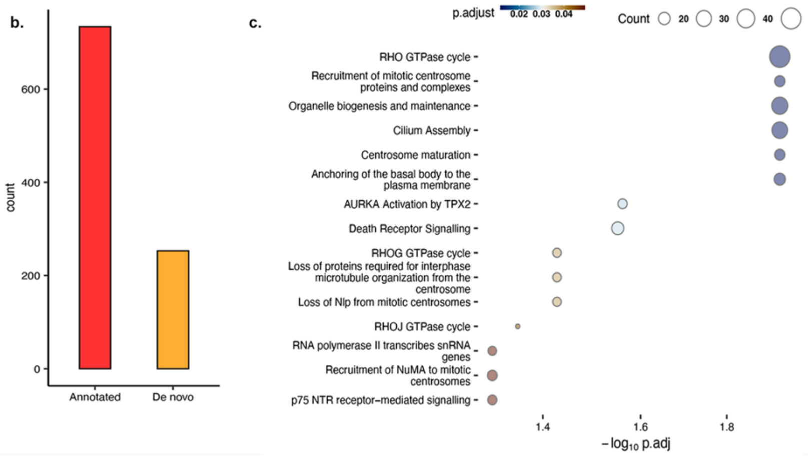

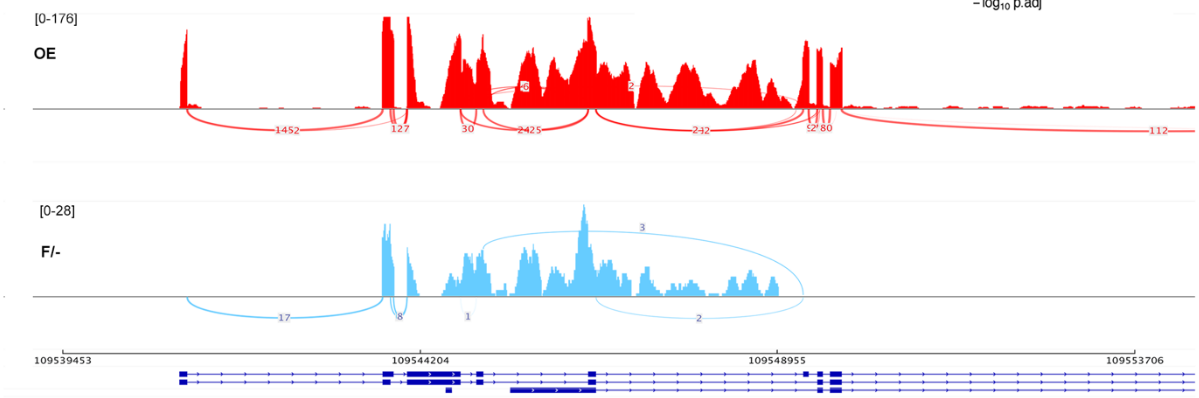

e.

Gene	junction coordinates	OE mean Ψ	FLX mean Ψ	mean ΔΨ	probability changing
Meg3	chr12:109544742-109544948	0.1194	0.0125	-0.0974	7.021e-09
Meg3	chr12:109544742-109546430	0.1767	0.1377	-0.0240	1.387e-09
Meg3	chr12:109544742-109549291	0.1050	0.0192	-0.0837	2.786e-20
Meg3	chr12:109544742-109549479	0.0563	0.0006	-0.0274	1.194e-20
Meg3	chr12:109544743-109544947	0.5419	0.8280	0.2910	9.729e-01
Rian	chr12:109645926-109646607	0.1030	0.4710	0.3720	9.920e-01
Rian	chr12:109645926-109649173	0.0686	0.0749	0.0033	4.677e-06
Rian	chr12:109646030-109646129	0.8266	0.4319	-0.3857	9.929e-01

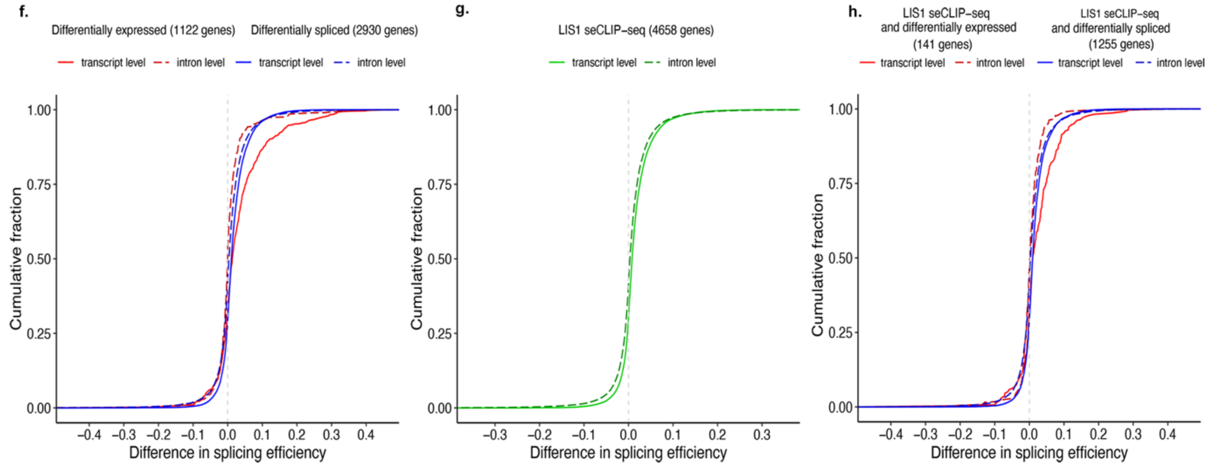

Supplementary Fig. 7: **LIS1 dosage affects RNA splicing. a**) A table summarizing differential alternative splicing events between ESCs lines overexpressing LIS-DsRED (LIS1 OE) and *Lis1*F/- (F/-) genotype identified with MAJIQ (by splice type) and RMATS. **b**) The number of annotated versus de novo events identified by MAJIQ. **c**) Pathway enrichment analysis of the genes that harbor differentially spliced events. The enrichment

analysis was performed using the Reactome database for genes found in MAJIQ and rMATS analyses using the clusterProfiler package in R. P-values for enriched GO terms were adjusted for multiple comparisons by the Benjamini-Hochberg method. **d**) Representative sashimi plots for the *Meg3* locus show differential splicing between the OE (red) and the F/- (light blue); the locus is shown at the bottom. **e**) A table summarizing splice events from MAJIQ (by deltapsi) for *Meg3* and *Rian* genes from *Meg3-Mirg* locus on mouse chromosome 12. **f-h**) An integrated transcript and intron-level quantification of splicing efficiency. **f)** Empirical cumulative distribution function (ECDF) plot for splicing efficiency differences in significant differentially expressed genes (1122 genes, red, two-sided paired Wilcoxon signed rank test, $p<0.05$) and genes with the significant differentially spliced events (2930 genes from MAJIQ, blue, two-sided paired Wilcoxon signed rank test, $p<0.05$) in OE and F/- comparison. **g**) ECDF plot for splicing efficiency differences in LIS1 bound (4658 genes from LIS1 seCLIP-seq, green, two-sided paired Wilcoxon signed rank test, $p<0.05$) in OE and F/- comparison. **h**) ECDF plot for splicing efficiency differences in overlapping LIS1 seCLIP-seq and differentially expressed genes (141 genes, red, $p<0.05$ two-sided paired Wilcoxon signed rank test), and overlapping LIS1 seCLIP-seq and genes with differentially spliced events (1255 genes, blue, $p<0.05$ two-sided paired Wilcoxon signed rank test) in OE and F/- comparison. In all ECDF plots, solid and dashed lines represent the differences between the transcript and intron-level splicing efficiency.

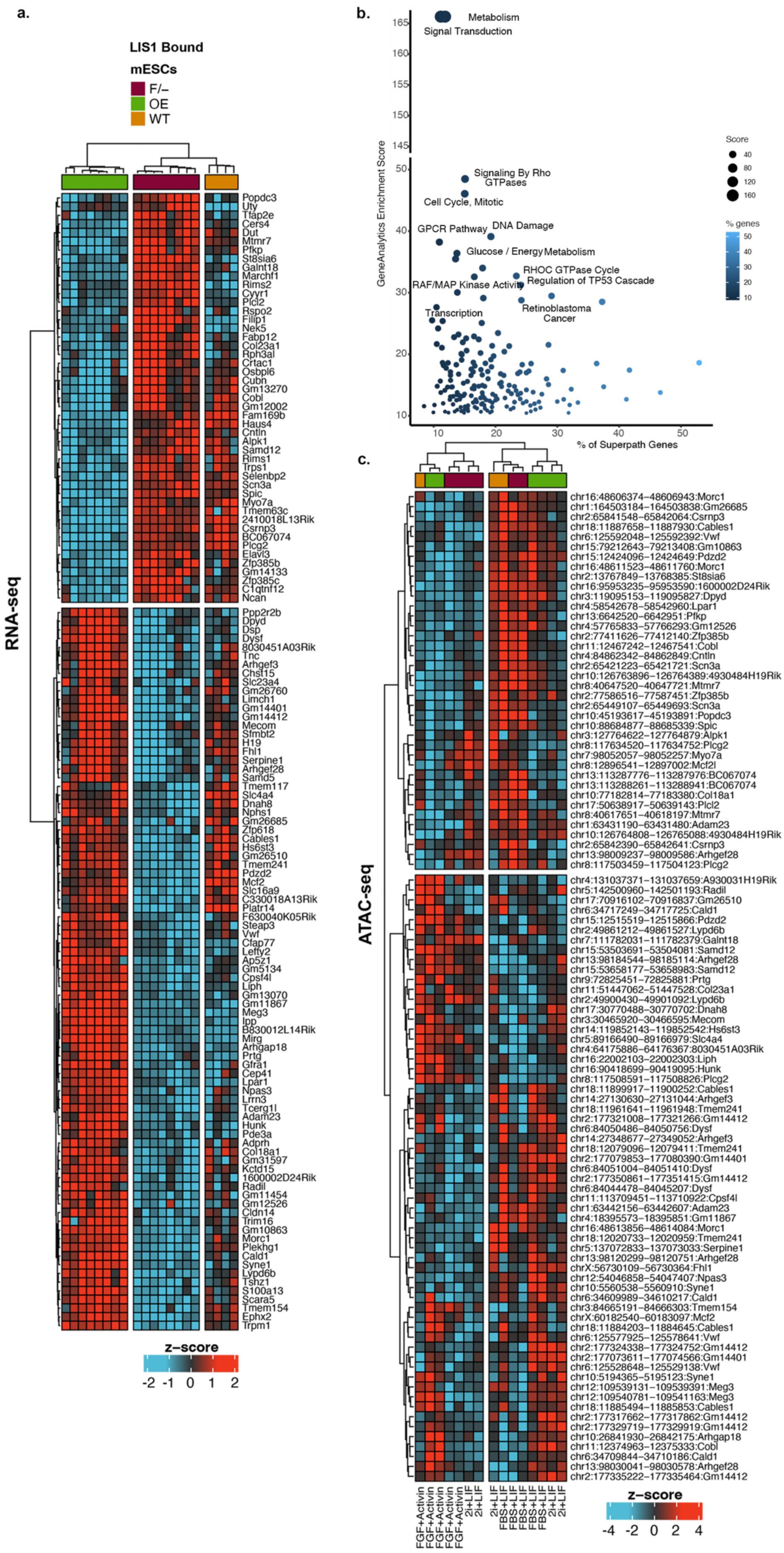

a.
LIS1 Bound
mESCs
F/–
OE
WT
RNA-seq
Popdc3
Uty
Tfap2e
Cers4
Dut
Mtmr7
Pfkp
St8sia6
Galnt18
Marchf1
Rims2
Cyyr1
Plcl2
Rspo2
Filip1
Nek5
Fabp12
Col23a1
Rph3al
Crtac1
Osbpl6
Cubn
Gm13270
Cobl
Gm12002
Fam169b
Haus4
Cntln
Alpk1
Samd12
Rims1
Trps1
Selenbp2
Scn3a
Spic
Myo7a
Tmem63c
2410018L13Rik
Csrnp3
BC067074
Plcg2
Elavl3
Zfp385b
Gm14133
Zfp385c
C1qtnf12
Ncan
Ppp2r2b
Dpyd
Dsp
Dysf
8030451A03Rik
Tnc
Arhgef3
Chst15
Slc23a4
Gm26760
Limch1
Gm14401
Gm14412
Mecom
Sfmbt2
H19
Fhl1
Serpine1
Arhgef28
Samd5
Tmem117
Slc4a4
Dnah8
Nphs1
Gm26685
Zfp618
Cables1
Hs6st3
Gm26510
Tmem241
Pdzd2
Mcf2
Slc16a9
C330018A13Rik
Platr14
F630040K05Rik
Steap3
Vwf
Cfap77
Lefty2
Ap5z1
Gm5134
Cpsf4l
Liph
Gm13070
Gm11867
Meg3
Ipp
B830012L14Rik
Mirg
Arhgap18
Prtg
Gfra1
Cep41
Lpar1
Npas3
Lrrn3
Tcerg1l
Adam23
Hunk
Pde3a
Adprh
Col18a1
Gm31597
Kctd15
1600002D24Rik
Radil
Gm11454
Gm12526
Cldn14
Trim16
Gm10863
Morc1
Plekhg1
Cald1
Syne1
Lypd6b
Tshz1
S100a13
Scara5
Tmem154
Ephx2
Trpm1
z–score
-2 -1 0 1 2
b.
Metabolism
Signal Transduction
Signaling By Rho GTPases
Cell Cycle, Mitotic
GPCR Pathway
DNA Damage
Glucose / Energy Metabolism
RHOC GTPase Cycle
RAF/MAP Kinase Activity
Regulation of TP53 Cascade
Transcription
Retinoblastoma Cancer
GeneAnalytics Enrichment Score
% of Superpath Genes
165
160
155
150
145
50
40
30
20
10
10 20 30 40 50
Score
40
80
120
160
% genes
50
40
30
20
10
c.
ATAC-seq
chr16:48606374–48606943:Morc1
chr1:164503184–164503838:Gm26685
chr2:65841548–65842064:Csrnp3
chr18:11887658–11887930:Cables1
chr6:125592048–125592392:Vwf
chr15:79212643–79213408:Gm10863
chr15:12424096–12424649:Pdzd2
chr16:48611523–48611760:Morc1
chr2:13767849–13768385:St8sia6
chr16:95953235–95953590:1600002D24Rik
chr3:119095153–119095827:Dpyd
chr4:58542678–58542960:Lpar1
chr13:6642520–6642951:Pfkp
chr4:57765833–57766293:Gm12526
chr2:77411626–77412140:Zfp385b
chr11:12467242–12467541:Cobl
chr4:84862342–84862849:Cntln
chr2:65421223–65421721:Scn3a
chr10:126763896–126764389:4930484H19Rik
chr8:40647520–40647721:Mtmr7
chr2:77586516–77587451:Zfp385b
chr2:65449107–65449693:Scn3a
chr10:45193617–45193891:Popdc3
chr10:88684877–88685339:Spic
chr3:127764622–127764879:Alpk1
chr8:117634520–117634752:Plcg2
chr7:98052057–98052257:Myo7a
chr8:12896541–12897002:Mcf2l
chr13:113287776–113287976:BC067074
chr13:113288261–113288941:BC067074
chr10:77182814–77183380:Col18a1
chr17:50638917–50639143:Plcl2
chr8:40617651–40618197:Mtmr7
chr1:63431190–63431480:Adam23
chr10:126764808–126765088:4930484H19Rik
chr2:65842390–65842641:Csrnp3
chr13:98009237–98009586:Arhgef28
chr8:117503459–117504123:Plcg2
chr4:131037371–131037659:A930031H19Rik
chr5:142500960–142501193:Radil
chr17:70916102–70916837:Gm26510
chr6:34717249–34717725:Cald1
chr15:12515519–12515866:Pdzd2
chr2:49861212–49861527:Lypd6b
chr7:111782031–111782379:Galnt18
chr15:53503691–53504081:Samd12
chr13:98184544–98185114:Arhgef28
chr15:53658177–53658983:Samd12
chr9:72825451–72825881:Prtg
chr11:51447062–51447528:Col23a1
chr2:49900430–49901092:Lypd6b
chr17:30770488–30770702:Dnah8
chr3:30465920–30466595:Mecom
chr14:119852143–119852542:Hs6st3
chr5:89166490–89166979:Slc4a4
chr4:64175886–64176367:8030451A03Rik
chr16:22002103–22002303:Liph
chr16:90418699–90419095:Hunk
chr8:117508591–117508826:Plcg2
chr18:11899917–11900252:Cables1
chr14:27130630–27131044:Arhgef3
chr18:11961641–11961948:Tmem241
chr2:177321008–177321266:Gm14412
chr6:84050486–84050756:Dysf
chr14:27348677–27349052:Arhgef3
chr18:12079096–12079411:Tmem241
chr2:177079853–177080390:Gm14401
chr6:84051004–84051410:Dysf
chr2:177350861–177351415:Gm14412
chr6:84044478–84045207:Dysf
chr11:113709451–113710922:Cpsf4l
chr1:63442156–63442607:Adam23
chr4:18395573–18395851:Gm11867
chr16:48613856–48614084:Morc1
chr18:12020733–12020959:Tmem241
chr5:137072833–137073033:Serpine1
chr13:98120299–98120751:Arhgef28
chrX:56730109–56730364:Fhl1
chr12:54046858–54047407:Npas3
chr10:5560538–5560910:Syne1
chr6:34609989–34610217:Cald1
chr3:84665191–84666303:Tmem154
chrX:60182540–60183097:Mcf2
chr18:11884203–11884645:Cables1
chr6:125577925–125578641:Vwf
chr2:177324338–177324752:Gm14412
chr2:177073611–177074566:Gm14401
chr6:125528648–125529138:Vwf
chr10:5194365–5195123:Syne1
chr12:109539131–109539391:Meg3
chr12:109540781–109541163:Meg3
chr18:11885494–11885853:Cables1
chr2:177317662–177317862:Gm14412
chr2:177329719–177329919:Gm14412
chr10:26841930–26842175:Arhgap18
chr11:12374963–12375333:Cobl
chr6:34709844–34710186:Cald1
chr13:98030041–98030578:Arhgef28
chr2:177335222–177335464:Gm14412
FGF+Activin
FGF+Activin
FGF+Activin
FGF+Activin
FGF+Activin
2i+LIF
2i+LIF
2i+LIF
FBS+LIF
FBS+LIF
FBS+LIF
FBS+LIF
FBS+LIF
2i+LIF
2i+LIF
z–score
-4 -2 0 2 4

Supplementary Fig. 8**: Gene expression and chromatin accessibility changes of LIS1 bound genes.** **a**) A heatmap showing the transcriptional changes between LIS1-DsRED overexpression (OE) and *Lis1* F/- (F/-) with wildtype (WT) mESCs in FBS+LIF for 134 LIS1 bound in seCLIP-seq and differentially expressed genes in RNAseq data. The data are shown on a Z-score scale of the variance stabilizing transformation on normalized reads. **b**) GeneAnalytics pathway enrichment analysis for a subset of differentially expressed genes between F/- ERT2 +4OHT (treated with tamoxifen) and F/- ERT2 LIS1-OE (with overexpression of LIS1) overlapping with LIS1 bound genes from LIS1 seCLIP-seq (n=1627). Top significant pathways identified by GeneAnalytics above an enrichment score of twenty for matched genes in Superpath are shown in the plot. **c**). A heatmap showing differential accessibility of 79 open chromatin regions associated with differentially expressed and LIS1-bound genes. The comparison is shown for OE and F/- transition across naïve and primed states (2i+LIF, FBS+LIF, and FGF+Activin). The data are shown on a Z-score scale of the variance stabilizing transformation on normalized primary alignment counts.

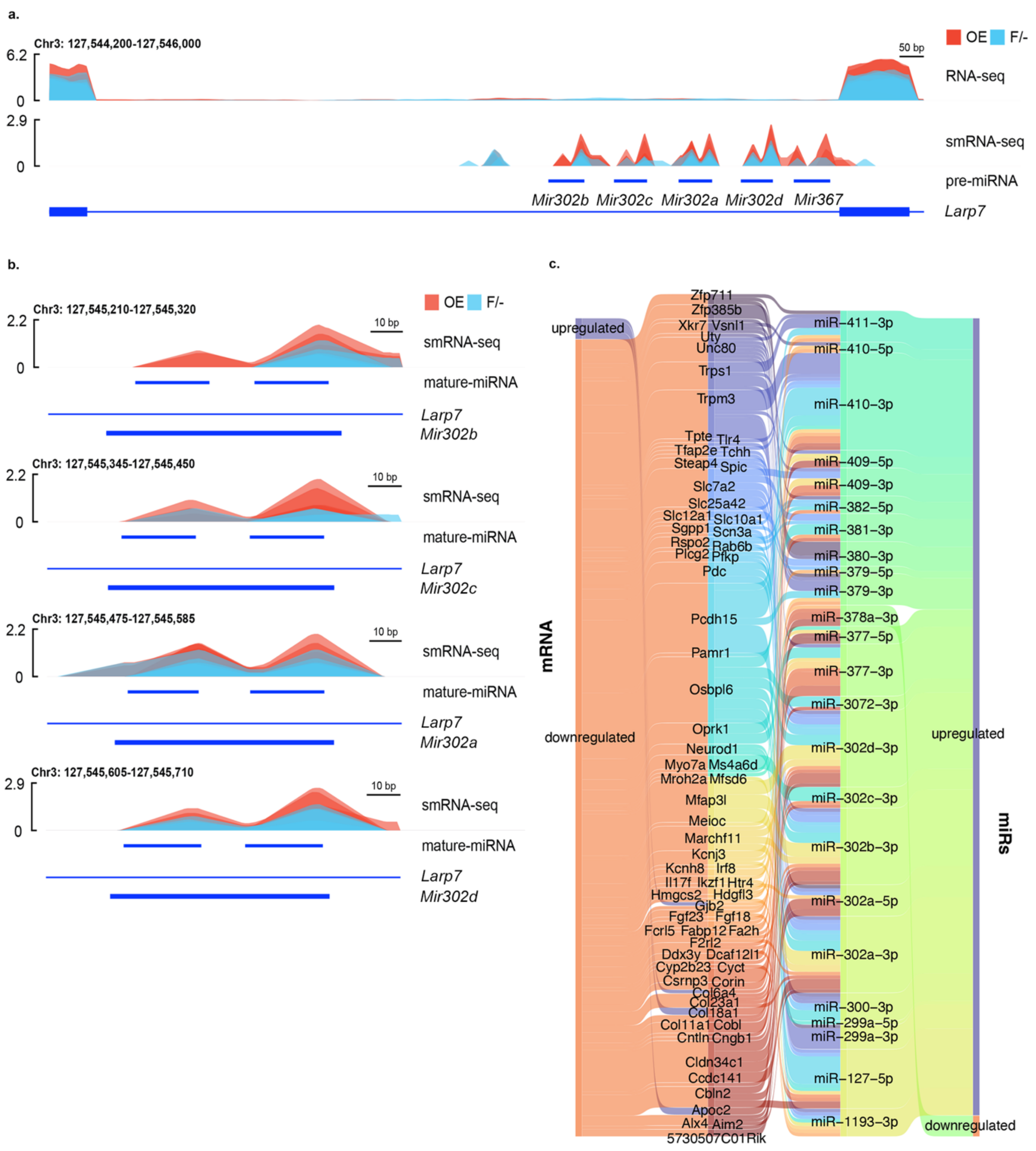

Supplementary Fig. 9: **LIS1 affects the expression of miRs and target genes. a**) Read coverage of small RNA-seq and total RNA-seq in the miR302-367 cluster (LIS1-DsRED overexpression (OE) in red and *Lis1* F/- (F/-) in light blue). **b**) Small RNA-seq read coverage of miR302b, miR302c, miR302a, and miR302d (top to bottom). OE in red and F/- in blue. **c**) Sankey diagram showing miRNA target analysis. Right: differentially expressed miRNAs between OE and F/- conditions. Left: differentially expressed genes between the same conditions. The miRNA targets were identified using the miRDB database[4]; only miRNA-RNA pairs with opposite expression change were selected.

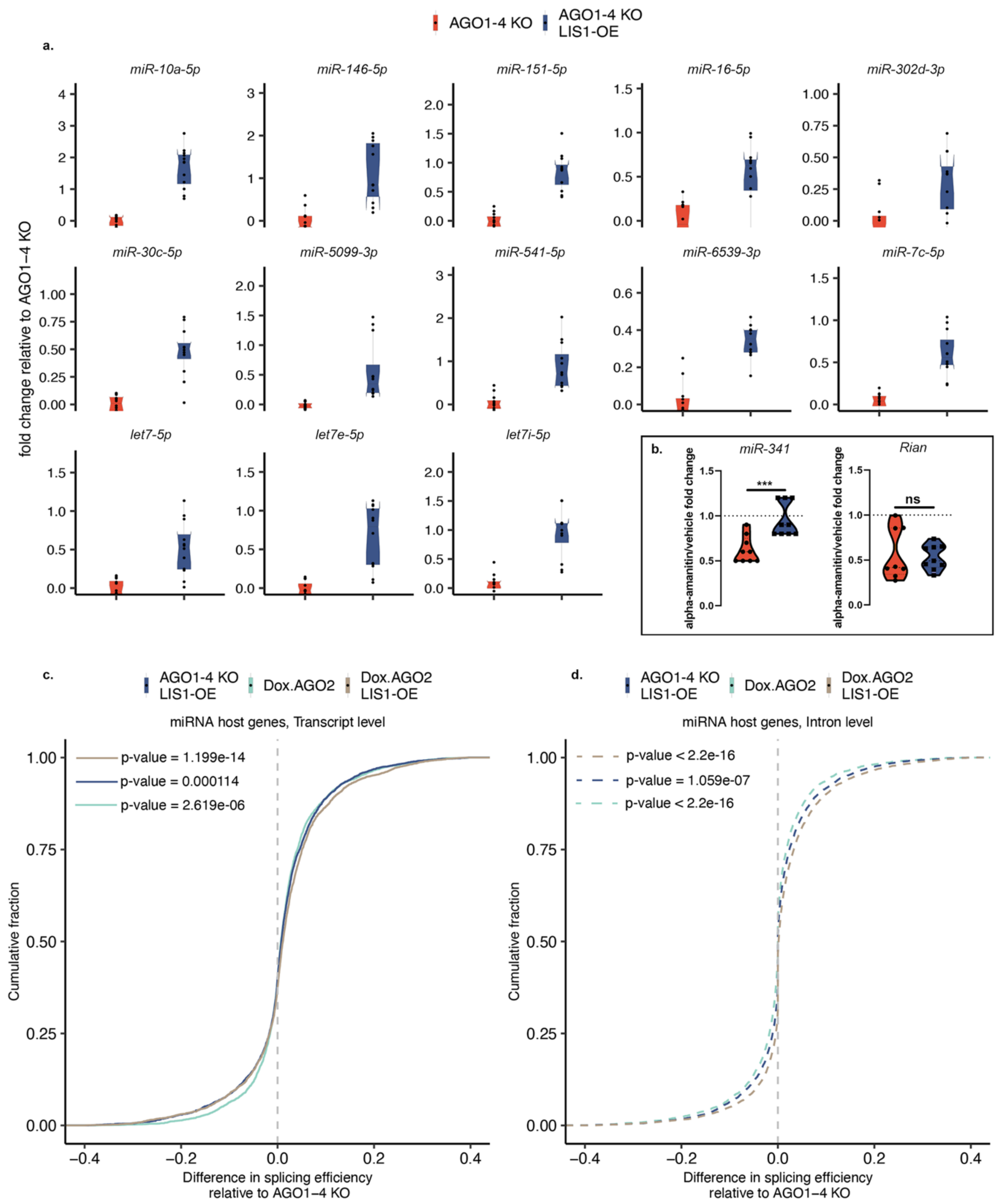

Supplementary Fig. 10**: LIS1 OE affects expression miRs and processing of miR host transcripts in AGO1-4 KO mESCs. a**) qRT-PCR validation of a subset of mature miRs that were found to be upregulated by overexpression of LIS1 on the background absence of all Argonaute proteins (AGO1-4 KO, red AGO1-4 KO LIS1 OE, blue). Boxplots show median and lower or upper quartiles; whiskers show inner fences. (n = 4 (x3), all p-values are reported in Supplementary Data 7E). **b**) qRT-PCR of *miR-341* and *Rian* in AGO 1-4 KO before (red) or three hours after treatment with the RNA polymerase II inhibitor, alpha-

amanitin (blue); *Rian*, p-value=0.95, miR-341, p-value=0.00028750, two-sided unpaired t-test was performed. **c**) Transcript level quantification of differences in splicing efficiency in miRNA host genes for Dox. AGO2 (light green), and Dox. AGO2 LIS1 OE (blue), and LIS1-OE in the absence of all AGO proteins AGO1-4 KO LIS1-OE; two-sided Kolmogorov-Smirnov test. **d**) Intron-level quantification of differences in splicing efficiency for indicated genotypes relative to AGO1-4KO in miRNA host genes; two-sided Kolmogorov-Smirnov test.

a. MAJIQ	Alternative first exon	Alternative Intron	Alternative last exon	Alternative 3' & 5'-ss	Alternative 3'-ss	Alternative 5'-ss	Cassette exon	Mutually exclusive exon	other	putative alternative 3' or 5'-ss	putative alternative first exon	putative alternative last exon
AGO1-4 KO LIS1 OE vs. AGO1-4KO	25	2	11	4	23	16	88	2	29	-	13	1
AGO1-4 KO LIS1 OE vs. Dox.AGO2	55	18	29	8	39	45	182	2	63	1	26	9
Dox.AGO2 LIS1 OE vs. AGO1-4 KO LIS1 OE	26	6	11	2	20	24	98	2	31	-	14	3
Dox.AGO2 LIS1 OE vs. AGO1-4KO	31	12	20	3	30	21	118	3	31	1	19	6
Dox.AGO2 LIS1 OE vs. Dox.AGO2	41	10	17	8	31	27	142	2	42	1	17	5
Dox.AGO2 vs. AGO1-4KO	46	12	20	7	40	39	171	4	47	1	28	5

b.

rMATS	Alternative 3'-SS	Alternative 5'-SS	Mutually exclusive exon	Retained intron	Skipped exon
AGO1-4 KO LIS1 OE vs. AGO1-4KO	286	179	129	330	738
AGO1-4 KO LIS1 OE vs. Dox.AGO2	300	220	244	420	952
Dox.AGO2 LIS1 OE vs. AGO1-4 KO LIS1 OE	246	204	154	324	826
Dox.AGO2 LIS1 OE vs. AGO1-4KO	287	191	137	304	799
Dox.AGO2 LIS1 OE vs. Dox.AGO2	241	175	184	327	763
Dox.AGO2 vs. AGO1-4KO	306	186	201	413	833

c.

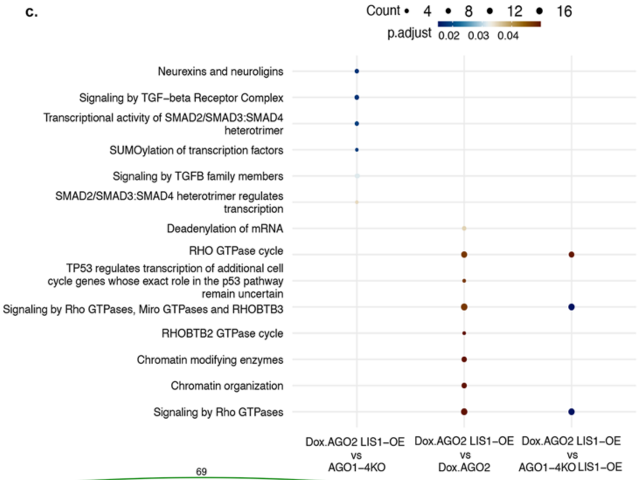

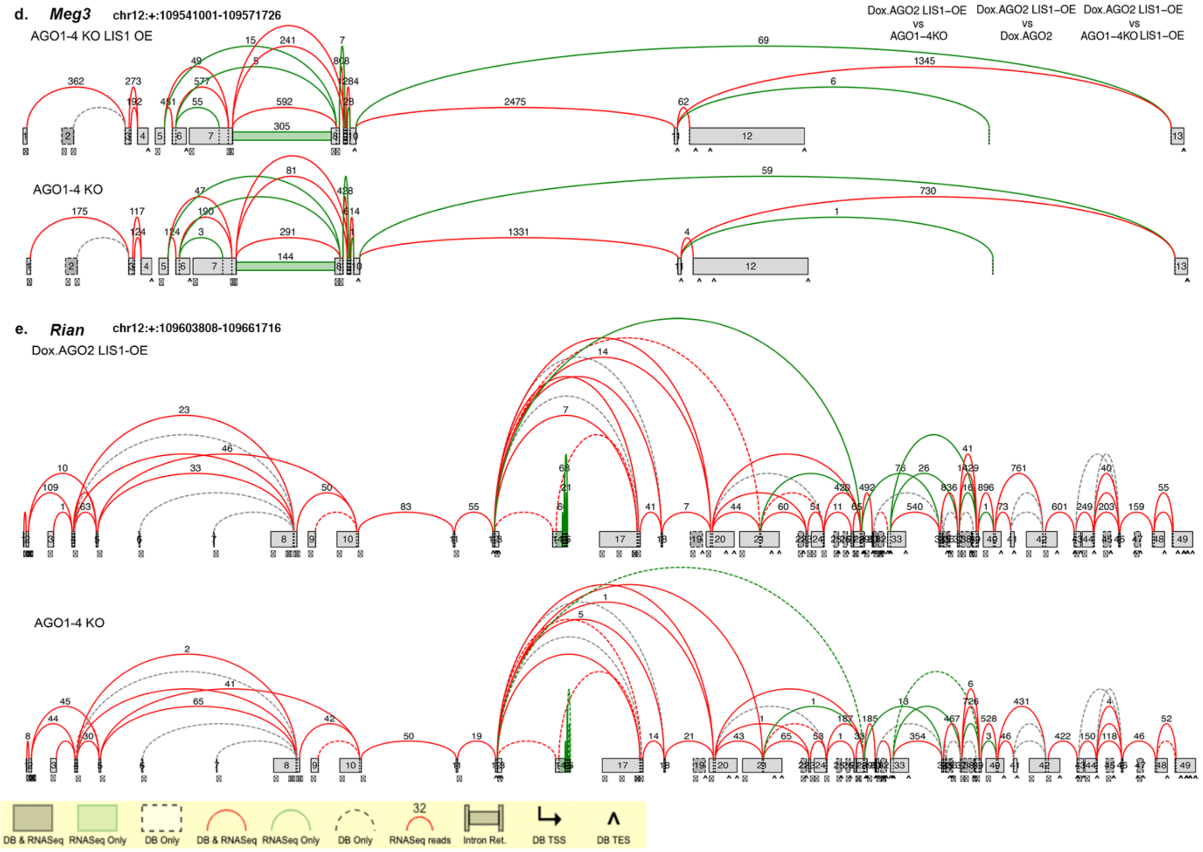

Supplementary Fig. 11**: LIS1 and AGO2 effects on RNA splicing. a-b**). Tables of all differential alternative splicing events identified with MAJIQ and RMATS between the following genotypes: AGO1-4 KO, Dox. AGO2, Dox.AGO2 LIS1-OE. **c).** Pathway enrichment analysis of the genes that harbor differentially spliced events. The analysis was

performed with the genes found in both MAJIQ and rMATS analysis, using the Reactome database to compare Dox.AGO2 LIS1-OE and AGO1-4 KO, Dox.AGO2 LIS1-OE and Dox.AGO2, and Dox.AGO2 LIS1-OE and LIS1-OE. **d**) Splice graphs for *Meg3* comparing LIS1-OE and AGO1-4 KO lines. **e**) Splice graphs for *Rian* comparing Dox.AGO2 LIS1-OE and AGO1-4 KO.

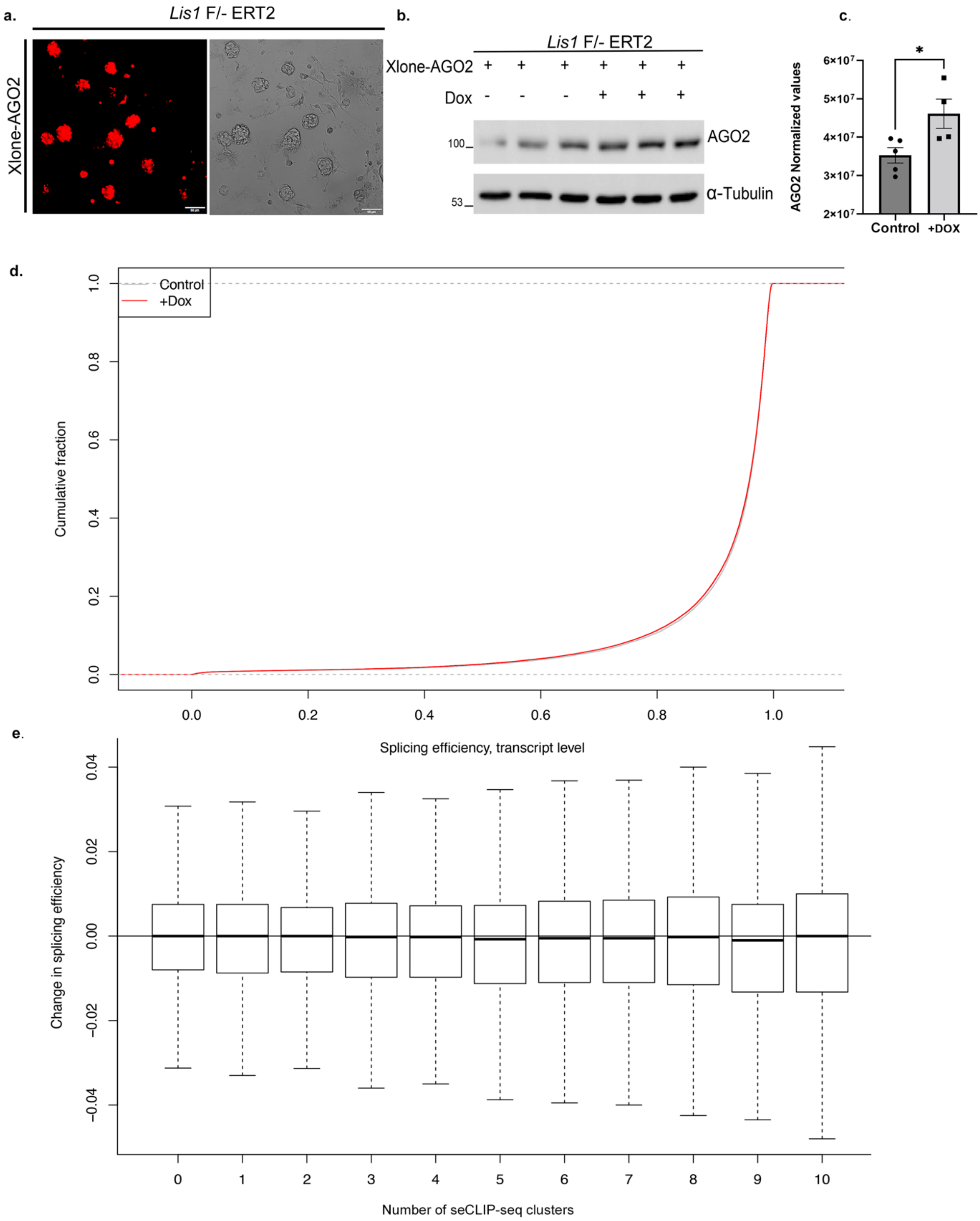

Supplementary Fig. 12: **AGO2 does not alter splicing efficiency in *Lis1* F/- cells.**

a) Bright field and fluorescence images of Xlone-AGO2, a tetracycline-inducible AGO2 line, on the background of LIS1 F/- ERT2, treated with Doxycycline (Dox). **b**-**c**) Expression analysis of AGO2 expression following Dox treatment in the Xlone-AGO2 clones by western blot analysis (**b**). **c**) AGO2 expression was normalized against α-Tubulin expression, showing a significant upregulation following Dox treatment (n=5), two-sided unpaired t-test, p=0.0316. **d**) Analysis of the RNA-seq data showed no difference in the cumulative fraction of the splicing efficiency at the transcript level before Dox treatment (gray line) and after DOX treatment of Xlone-AGO2 lines (red line). For the rescue on the transcript level, for the transcript-level comparison, n=64,303, and the difference is actually statistically significant by Wilcoxon rank-sum test, two-sided, P<10^(-16), but it is very small, the median difference is 0.000647, and the mean difference is 0.00082. **e**) No effect was noted on the splicing efficiency in relation to the number of LIS1 seCLIP-seq clusters, following AGO2 induction, in Xlone-AGO2 clones (n=4). P>0.2 for comparisons within each group with a two-sided Wilcoxon rank-sum test, and n=15659, 7471, 6579, 4950, 4127, 3294, 2667, 2156, 1714, 1397, 12004 (for the 11 groups). Boxplots show median and lower or upper quartiles; whiskers show inner fences.

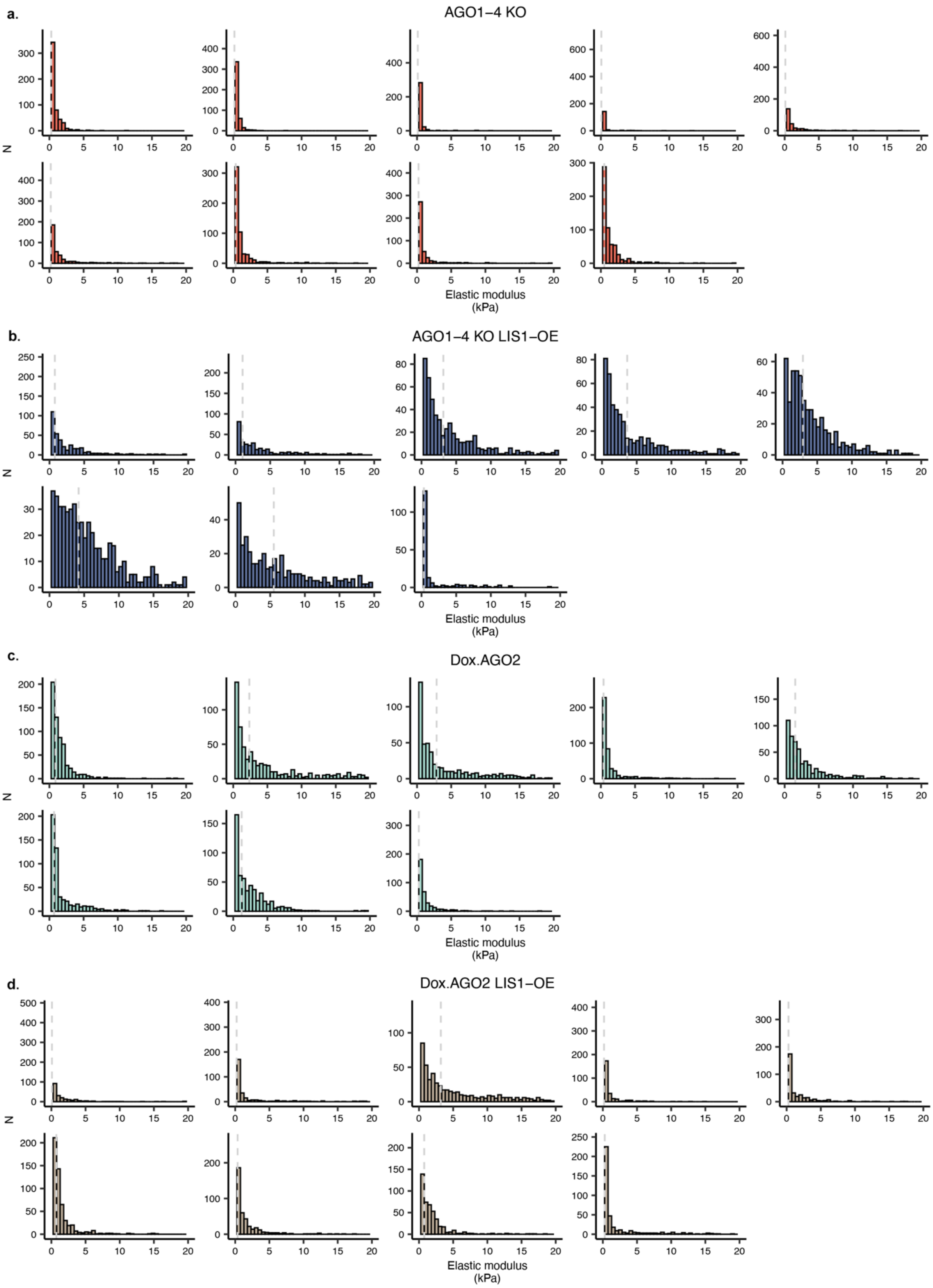

Supplementary Fig. 13**: LIS1 overexpression affects the elastic modulus of AGO1-4 KO mESCs.** Elastic modulus histograms for embryonic stem-cell colonies. **a.** AGO1-4 KO (n=9). **b.** AGO1-4 KO over expressing LIS1-GFP (LIS1-OE, n=8). **c.** Doxycycline (DOX) treated

AGO1-4 KO lines with Tet-On Argonaute 2 expression (Dox.AGO2, (n=8). **d.** DOX treated AGO1-4 KO over expressing LIS1-GFP (Dox.AGO2 LIS1-OE, n=8).

Supplementary References

1. Guruharsha, K.G., Rual, J.F., Zhai, B., Mintseris, J., Vaidya, P., Vaidya, N., Beekman, C., Wong, C., Rhee, D.Y., Cenaj, O., et al. (2011). A protein complex network of Drosophila melanogaster. Cell *147*, 690-703. 10.1016/j.cell.2011.08.047.
2. Sarshad, A.A., Juan, A.H., Muler, A.I.C., Anastasakis, D.G., Wang, X., Genzor, P., Feng, X., Tsai, P.F., Sun, H.W., Haase, A.D., et al. (2018). Argonaute-miRNA Complexes Silence Target mRNAs in the Nucleus of Mammalian Stem Cells. Mol Cell *71*, 1040-1050 e1048. 10.1016/j.molcel.2018.07.020.
3. Leung, A.K., Young, A.G., Bhutkar, A., Zheng, G.X., Bosson, A.D., Nielsen, C.B., and Sharp, P.A. (2011). Genome-wide identification of Ago2 binding sites from mouse embryonic stem cells with and without mature microRNAs. Nature structural & molecular biology *18*, 237-244. 10.1038/nsmb.1991.
4. Chen, Y., and Wang, X. (2020). miRDB: an online database for prediction of functional microRNA targets. Nucleic Acids Res *48*, D127-D131. 10.1093/nar/gkz757.
