# Supplementary material for: LIS1 RNA-binding orchestrates the mechanosensitive properties of embryonic stem cells in AGO2-dependent and independent ways
Source: Nat Commun. 2023 Jun 6;14:3293. doi: 10.1038/s41467-023-38797-8 (PMC10244377; doi:10.1038/s41467-023-38797-8)
Supplement: Supplementary file 3 — Description of Additional Supplementary Files [file 41467_2023_38797_MOESM3_ESM.pdf]

Description of Additional Supplementary Files

File Name: Supplementary Data 1
Description: **a.** DESeq2 results from MARS-seq experiment for differential expression of genes. Comparison between F/- ErT2 (+4OHT), F/- ErT2, F/- Ert2 LIS1OE (+4OHT) and F/- Ert2 LIS1OE(GFP) in 5i+LIF condition.

File Name: Supplementary Data 2
Description: **a-e.** Spectral intensities, quantification, and statistics of polar metabolites from F/-, WT, and LIS1-OE(DsRed) mESCs in Serum+LIF condition. **f-i.** Spectral intensities, quantification, and statistics of fatty acid metabolites from F/-, WT, and LIS1-OE(DsRed) mESCs in Serum+LIF condition.

File Name: Supplementary Data 3
Description: **a.** DESeq2 results from MARS-seq experiment for differential expression of genes. Comparison between LIS1 +/-, Control(WIBR3), LIS1 ex6*/*, and LIS1-OE(GFP) in NHSM and tHENSM human naive pluripotency conditions **b.** The GeneAnalytics pathway enrichment analysis for differentially expressed genes(n=897) between LIS1 +/- and LIS1-OE(GFP). **c.** Common differentially expressed genes between human and mouse LIS1 dosage lines.

File Name: Supplementary Data 4
Description: **a.** Label-free peptide intensities and the number of razor+unique peptides for proteins identified by mass spectrometry after LIS1 immunoprecipitation (IP) in the cytoplasm and nuclear lysates of LIS1 F/-, WT, and LIS1-OE(DsRED) mESCs in Serum+LIF condition. **b.** Label-free peptide intensities and the number of razor+unique peptides for proteins identified by mass spectrometry after LIS1 immunoprecipitation in the whole cell extracts of AGO1-4 KO and Dox. AGO2 (doxycycline induced expression of AGO2) mESCs in Serum+LIF condition. **c.** The total number of proteins in the LIS1 interactome **d.** List of RNA binding proteins from IP-LIS1 mass spectrometry dataset and their interacting proteins from BioGRID. **e.** Percent and significance for RNA binding protein interactome and LIS1 protein network overlap.

File Name: Supplementary Data 5
Description: Results from MAJIQ and RMATS for Lis1 mESCs derived from blastocysts. **a-b.** MAJIQ LSVs and splice events for LIS1 OE and F/-. **c.** RMATS splice events for LIS1 OE and F/-. **d-e.** MAJIQ LSVs and splice events for WT and F/-. **f.** RMATS splice events for WT and F/-.

File Name: Supplementary Data 6
Description: Statistics for **a.** Supplementary figure 6b-c. **b.** Figure 3f. **c.** Figure 4e **d.** Fig.5c-e and Supplementary figure 8a. **e.** Supplementary figure 10c-d. **f.** Figure 6d. **g.** Figure 6f.

File Name: Supplementary Data 7
Description: **a.** LIS1 seCLIP-seq peaks from CLIPper. **b.** A subset of differentially expressed genes between F/- ERT2 +4OHT (treated with tamoxifen) and F/- ERT2 LIS1-OE (with overexpression of LIS1) mESCs overlapping with LIS1 bound genes from LIS1 seCLIP-seq. **c.** GeneAnalytics enrichment analysis for (b). **d.** The difference in mean transcript ratios for LIS1 bound genes in LIS1 OE vs. F/- and WT vs. F/- mESCs comparisons. **e.** A subset of genes with change in splicing outcome from MAJIQ between OE and F/- mESCs overlapping with LIS1 bound genes from LIS1 seCLIP-seq.

File Name: Supplementary Data 8
Description: **a.** ATACseq differentially accessible chromatin regions between F/-, WT, and OE mESCs in 2i+LIF, Serum+LIF, and FGF+Activin conditions. **b.** Transcription factor footprinting for differentially accessible regions.

File Name: Supplementary Data 9
Description: **a.** DESeq2 results from small RNAseq experiment for differential expression of miRs. Comparison between F/-, WT, and OE mESCs in serum+LIF condition.

File Name: Supplementary Data 10
Description: **a.** DESeq2 results from total RNAseq experiment for differential expression of genes. Comparison between F/-, WT, and OE mESCs in serum+LIF condition.

File Name: Supplementary Data 11
Description: **a.** DESeq2 results from total RNAseq experiment for differential expression of miRs. Comparison between AGO1-KO, Dox.AGO2, AGO1-4 KO LIS1-OE, Dox.AGO2 LIS1-OE mESCs in serum+LIF condition.

File Name: Supplementary Data 12
Description: **a.** DESeq2 results from total RNAseq experiment for differential expression of genes. Comparison between AGO1-KO, Dox.AGO2, AGO1-4 KO LIS1-OE, Dox.AGO2 LIS1-OE mESCs in serum+LIF condition. **b.** The GeneAnalytics pathway enrichment analysis for differentially expressed genes between AGO1-4 KO and AGO1-4KO LIS1-OE.

File Name: Supplementary Data 13
Description: Results from MAJIQ and RMATS for AGO1-4 KO derived mESCs. **a.** MAJIQ LSVs and splice events for AGO1-4 KO and Dox. AGO2. **b.** MAJIQ LSVs and splice events for AGO1-4 KO and AGO1-4 KO LIS1-OE. **c.** MAJIQ LSVs and splice events for AGO1-4 KO and Dox. AGO2 LIS1-OE. **d.** MAJIQ LSVs and splice events for AGO1-4 KO LIS1-OE and Dox. AGO2. **e.** MAJIQ LSVs and splice events for Dox. AGO2 LIS1-OE and Dox. AGO2. **f.** MAJIQ LSVs and splice events for AGO1-4 KO LIS1- OE and Dox. AGO2 LIS1-OE. **g.** MAJIQ LSVs and splice events combined for all the comparisons. **h.** RMATS splice events for all the comparisons
