# Supplementary material for: LIS1 RNA-binding orchestrates the mechanosensitive properties of embryonic stem cells in AGO2-dependent and independent ways
Source: Nat Commun. 2023 Jun 6;14:3293. doi: 10.1038/s41467-023-38797-8 (PMC10244377; doi:10.1038/s41467-023-38797-8)
Supplement: Supplementary file 17 — Reporting Summary [file 41467_2023_38797_MOESM17_ESM.pdf]

nature portfolio

Corresponding author(s): Orly Reiner

Last updated by author(s): Apr 30, 2023

Reporting Summary

Nature Portfolio wishes to improve the reproducibility of the work that we publish. This form provides structure for consistency and transparency in reporting. For further information on Nature Portfolio policies, see our Editorial Policies and the Editorial Policy Checklist.

Statistics

For all statistical analyses, confirm that the following items are present in the figure legend, table legend, main text, or Methods section.

n/a	Confirmed	
☐	☒	The exact sample size (*n*) for each experimental group/condition, given as a discrete number and unit of measurement
☐	☒	A statement on whether measurements were taken from distinct samples or whether the same sample was measured repeatedly
☐	☒	The statistical test(s) used AND whether they are one- or two-sided<br>*Only common tests should be described solely by name; describe more complex techniques in the Methods section.*
☒	☐	A description of all covariates tested
☐	☒	A description of any assumptions or corrections, such as tests of normality and adjustment for multiple comparisons
☐	☒	A full description of the statistical parameters including central tendency (e.g. means) or other basic estimates (e.g. regression coefficient) AND variation (e.g. standard deviation) or associated estimates of uncertainty (e.g. confidence intervals)
☐	☒	For null hypothesis testing, the test statistic (e.g. *F*, *t*, *r*) with confidence intervals, effect sizes, degrees of freedom and *P* value noted<br>*Give P values as exact values whenever suitable.*
☒	☐	For Bayesian analysis, information on the choice of priors and Markov chain Monte Carlo settings
☐	☒	For hierarchical and complex designs, identification of the appropriate level for tests and full reporting of outcomes
☒	☐	Estimates of effect sizes (e.g. Cohen's *d*, Pearson's *r*), indicating how they were calculated

Our web collection on statistics for biologists contains articles on many of the points above.

Software and code

Policy information about availability of computer code

Data collection	No new software was generated for this study.<br>Source code and software availability:<br>UTAP pipeline - https://bitbucket.org/bbcu/utap (v1.10).<br>ATAC seq pipelines - https://github.com/kundajelab/atac_dnase_pipelines and https://github.com/loosolabiTOBIAS<br>eCLIP pipeline- https://github.com/YeoLab/merge_peaks<br>Maxquant- https://github.com/JurgenCox/compbio-base
Data analysis	MAJIQ- https://biociphers.bitbucket.io/majiq/(v2.1)<br>MOCCASIN- https://bitbucket.org/biociphers/moccasin/src/master/<br>RMATS- https://rnaseq-mats.sourceforge.net/rmats4.1.1/download.html (v4.1.1)<br>deepTools- https://github.com/deeptools/deepTools<br>ngsplot- https://github.com/shenlab-sinai/ngsplot<br>SparK- https://github.com/harbourlab/SparK<br>homer/4.1<br>Following R and bioconductor packages were used to perform data analysis in R (v4.1.2)<br>AnnotationDbi_1.56.2, Biobase_2.54.0, BiocGenerics_0.40.0, BiocParallel_1.28.0, biomaRt_2.50.1, Biostrings_2.62.0, BSgenome_1.62.0, clusterProfiler_4.2.2, ComplexHeatmap_2.10.0, DEP_1.16.0, DESeq2_1.34.0, dplyr_1.0.8, edgeR_3.36.0, GencoDymo_0.6.1, genefilter_1.76.0, genomation_1.26.0, GenomeInfoDb_1.30.0, GenomicFeatures_1.46.1, GenomicRanges_1.46.1, ggplot2_3.4.1, ggrepel_0.9.1, Gviz_1.38.4, IRanges_2.28.0, limma_3.50.3, plyranges_1.14.0, purrr_1.0.1, QuasR_1.34.0, ReactomePA_1.38.0, Rsamtools_2.10.0, rtracklayer_1.54.0,

rstatix_0.7.2, statsExpressions_1.4.0, stringr_1.5.0, sva_3.42.0, tidyr_1.3.0, tidyverse_1.3.1, valr_0.6.3, viridis_0.6.2, viridisLite_0.4.1.
Gencodymo- https://github.com/monahton/GencoDymo and https://zenodo.org/record/3605996#.ZEzyqOxBzdo

For manuscripts utilizing custom algorithms or software that are central to the research but not yet described in published literature, software must be made available to editors and reviewers. We strongly encourage code deposition in a community repository (e.g. GitHub). See the Nature Portfolio guidelines for submitting code & software for further information.

Data

Policy information about availability of data

All manuscripts must include a data availability statement. This statement should provide the following information, where applicable:

- Accession codes, unique identifiers, or web links for publicly available datasets
- A description of any restrictions on data availability
- For clinical datasets or third party data, please ensure that the statement adheres to our policy

All sequencing data generated have been deposited in the GEO database and is available under accession code GSE198390.
The mass spectrometry data has been submitted in the ProteomeXchange database and is available under accession code is PXD033150.
GRCm38/mm10 - https://ftp.ebi.ac.uk/pub/databases/gencode/Gencode_mouse/release_M25/GRCm38.p6.genome.fa.gz
GRCh38/hg38 - https://ftp.ebi.ac.uk/pub/databases/gencode/Gencode_human/release_38/GRCh38.p13.genome.fa.gz

Research involving human participants, their data, or biological material

Policy information about studies with human participants or human data. See also policy information about sex, gender (identity/presentation), and sexual orientation and race, ethnicity and racism.

Reporting on sex and gender	N/A
Reporting on race, ethnicity, or other socially relevant groupings	N/A
Population characteristics	N/A
Recruitment	N/A
Ethics oversight	N/A

Note that full information on the approval of the study protocol must also be provided in the manuscript.

Field-specific reporting

Please select the one below that is the best fit for your research. If you are not sure, read the appropriate sections before making your selection.

☒ Life sciences ☐ Behavioural & social sciences ☐ Ecological, evolutionary & environmental sciences

For a reference copy of the document with all sections, see nature.com/documents/nr-reporting-summary-flat.pdf

Life sciences study design

All studies must disclose on these points even when the disclosure is negative.

Sample size	No statistical tests or explicit calculations were performed to determine sample size. For the sample size and the number of repetitions for each experiment, we referred to the design in the most published studies.
Data exclusions	No data were excluded in the analysis.
Replication	The main dataset involving total RNA or small RNA sequencing generated in the paper contain four biological replicates for each embryonic stem cell lines reported. Following standard practice for eCLIP and ATAC sequencing two biological replicates were used. For mass spectrometry data total two independent immunoprecipitations were performed for whole cell extract, nuclear and cytoplasmic fractions concerned with each genotype reported with respective controls. Other experimental findings are reliably reproduced with three biological replicates. All attempts at replication were successful.
Randomization	Randomization was not considered since this study does not involve cohorts of animals or human individuals. Comparison were done between cells from different genotypes or treated and control cells.
Blinding	Blinding was not considered for this study. All cells were grown in identical culture conditions (+/- treatment). No subjective measurements were applied.

Reporting for specific materials, systems and methods

We require information from authors about some types of materials, experimental systems and methods used in many studies. Here, indicate whether each material, system or method listed is relevant to your study. If you are not sure if a list item applies to your research, read the appropriate section before selecting a response.

Materials & experimental systems

n/a	Involved in the study
☐	☒ Antibodies
☐	☒ Eukaryotic cell lines
☒	☐ Palaeontology and archaeology
☐	☒ Animals and other organisms
☒	☐ Clinical data
☒	☐ Dual use research of concern
☒	☐ Plants

Methods

n/a	Involved in the study
☒	☐ ChIP-seq
☒	☐ Flow cytometry
☒	☐ MRI-based neuroimaging

Antibodies

Antibodies used

NANOG (A300-397A, Thermofisher): IF-1:200, WB 1:1000
OCT4 (2840, Cell Signaling): IF-1:200, WB 1:1000
OCT4A (C30A3, Cell Signaling): WB 1:1000, IF 1:200
OCT4 (H134, Santacruz): IF-1:200, WB 1:1000
LIS1 (338): IP-20u1, IF-1:200, WB-1:1000, eCLIP-30ul
E-CADHERIN (ab1416, Abcam) :IF 1:200
Pan-LEFTY (ab22569, Abcam):IF 1:200
Goat Anti-Mouse-HRP (111-035-144, Jackson labs): WB 1:10000
Goat Anti-Rabbit-HRP (ab97023, Jackson labs): WB 1:10000
Goat Anti-Rabbit-Cy3 (111-165-003, Jackson labs): IF 1:300
Donkey Anti-Mouse-Alexa Fluor® 488 (715-545-150, Jackson labs): IF 1:300
Donkey Anti-Mouse-Cy3 (715-165-150, Jackson labs): IF 1:300
Argonaute-2 (2E12-1C91, Abcam): WB 1:500
Argonaute-2 (11A9, Sigma Aldrich): IP 20ul
YAP/TAZ (D24E4, Cell Signaling): WB 1:1000
alpha-Tubulin (clone DM1A, sigma T9026): WB 1:1000
Lamin A/C (4c11, Cell Signaling): WB 1:1000
HSC70 (sc-7928, Santa Cruz) antibody: WB 1:1000
Beta-catenin (E247, Abcam) antibody: IF 1:200
NANOG (4903, Cell Signaling): WB 1:2000, IF 1:200

Validation

The antibodies were either verified by the companies or previously published manuscripts.
NANOG antibody: https://www.thermofisher.com/antibody/product/Nanog-Antibody-Polyclonal/A300-397A
OCT4A antibody (WB, ICC): https://www.cellsignal.com/products/primary-antibodies/oct-4a-c30a3-rabbit-mab/2840
OCT4 antibody: https://www.scbt.com/p/oct 3 4 antibody-h-134
LIS1 antibody(338): https://doi.org/10.1093/emboj/16.23.6977
E-CADHERIN antibody: https://www.abcam.com/e-cadherin-antibody-hecd-1-intercellular-junction-marker-ab1416.html
Pan-LEFTY antibody: https://www.abcam.com/lefty-antibody-ab22569.html
Argonaute-2 antibody (IP): https://www.sigmaaldrich.com/IL/en/product/mm/mabe253
Argonaute-2 antibody (WB): https://www.abcam.com/argonaute-2-antibody-2e12-1c9-bsa-and-azide-free-ab57113.html
YAP/TAZ antibody: https://www.cellsignal.com/products/primary-antibodies/yap-taz-d24e4-rabbit-mab/8418
Tubulin antibody: https://www.sigmaaldrich.com/IL/en/product/sigma/t9026
Lamin A/C antibody: https://www.cellsignal.com/products/primary-antibodies/lamin a c 4c11 mouse-mab/4777
HSC70 antibody: https://www.scbt.com/p/hsc-70-antibody-b-6
Beta-catenin antibody: https://www.abcam.com/beta-catenin-antibody-e247-chip-grade-ab32572.html
Goat Anti-Rabbit (HRP): https://www.jacksonimmuno.com/catalog/products/111-035-144
Goat Anti-Mouse (HRP): https://www.abcam.com/goat-mouse-igg-hl-hrp-ab97023.html
Goat Anti-Rabbit (Cy3):https://www.jacksonimmuno.com/catalog/products/111-165-003
Donkey Anti-Mouse(Alexa Fluor 488):https://www.jacksonimmuno.com/catalog/products/715-545-150
Donkey Anti-Mouse(Cy3):https://www.jacksonimmuno.com/catalog/products/715-165-150

Eukaryotic cell lines

Policy information about cell lines and Sex and Gender in Research

Cell line source(s)

Following mouse embryonic stem cell lines with LIS1 genotypes were derived from E3.5 blastocyst and are originated in our laboratory at the Weizmann Institute of Science:
LIS1 F/- ERT2, LIS1 F/- (two cell lines), Wild type (WT, hybrid), LIS1-OE(LIS1-DsRED overexpression, two cell lines).
AGO1-4KO (TT-FHAgo2) mouse embryonic stem cell line originated from Prof. Sharp Lab, MIT.

	V6.5 mouse embryonic stem cell line originated from Prof. Jaenisch Lab, MIT.<br>WIBR3 (NIHhESC-10-0079) human embryonic stem cell line originated from Prof. Jaenisch Lab, MIT.<br>Mouse embryonic fibroblast feeders(DR4) were either derived in our lab or Stem Cell Facility at the Weizmann Institute of Science.
Authentication	Genotype of cell lines was tested at the level of DNA sequence and protein.
Mycoplasma contamination	All cell lines were routinely tested and confirmed negative for mycoplasma.
Commonly misidentified lines<br>(See ICLAC register)	No cell lines used in this study were found in the register of Misidentified Cell Lines maintained by ICLAC (version 11).

Animals and other research organisms

Policy information about studies involving animals; ARRIVE guidelines recommended for reporting animal research, and Sex and Gender in Research

Laboratory animals	1129S-Pafah1b1tm2Awb/J [Lis1 F/F]- and B6;129S-Tg(UBC-cre/ERT2)1Ejb/J [ErT2-Cre] were purchased from Jackson Laboratory.<br>129S;ICR-Tg(CAGG-loxP-LacZ-neo-loxP-PAFAH1B1-DsRed) [LIS1-Flag-DsRed] generated at the Weizmann Institute of Science, DOI:https://doi.org/10.1038/ng.302<br>B6-Tg(Pgk1-cre)1Lni generated at the Weizmann Institute of Science, DOI: 10.1023/a:1008868325009<br><br>All the animals were bred and maintained at the Lokey preclinical research facility (MAMTAK) or the transgenic mice facility, Weizmann Institute of Science. The mice were kept in the animal facility at a temperature of 22°C±1°C, 50%±10% humidity, and a 12 h light/dark cycle.
Wild animals	The study did not involve wild animals.
Reporting on sex	Both sexes were used for breeding with ages over 8 weeks to flush blastocysts and derive embryonic stem cell lines. Except for B6-Tg(Pgk1-cre)1Lni mice, where only females were used considering maternal cre activation.
Field-collected samples	The study did not involve samples collected from the field.
Ethics oversight	All animal studies were approved by The Weizmann Institute's Institutional Animal Care and Use Committee (IACUC). Experiments using human stem cells were approved by Weizmann Institutional Review Board (IRB).

Note that full information on the approval of the study protocol must also be provided in the manuscript.
